# Anatomical description and digital reconstruction of the skull of *Jeholosaurus shangyuanensis* (Dinosauria, Ornithopoda) from China

**DOI:** 10.1371/journal.pone.0312519

**Published:** 2025-01-24

**Authors:** Filippo Bertozzo, Niu Kecheng, Nathan Vallée Gillette, Pascal Godefroit

**Affiliations:** 1 Institute of Natural Sciences, Brussels, Belgium; 2 Sociedade de Historia Natural, Torres Vedras, Portugal; 3 Yingliang Stone Natural History Museum, Nan’an, China; 4 State Key Laboratory of Cellular Stress Biology, School of Life Sciences, Xiamen, China; Chinese Academy of Sciences, CHINA

## Abstract

Ornithopod dinosaurs appeared during the Middle Jurassic, but it was in the Lower Cretaceous they started their successful evolutionary history. Different phylogenies describing the evolutionary relationships of Ornithopoda are mostly based on cranial features, however there is a lack of well-preserved and complete skulls for the basal member of the clade, hampering our knowledge on the mode and tempo of these herbivorous dinosaurs. Here we describe YLSNHM 01942, a well-preserved skull of a juvenile neornithischian from the Liaoning Province of China. The specimen was scanned with a μCT scan, and all the elements were segmented and extrapolated for description. The specimen shows a ventral deformation due to the compression of the sediment, and a few rostral elements were artificially added. The specimen is attributed to the basal ornithopod *Jeholosaurus shangyuanensis* because of the presence of a large foramen in the quadratojugal, however it lacks the nodular ornamentation on the postorbital and jugal, herein interpreted as an ontogenetic feature. This, together with the disarticulation degree of the cranial elements, suggest YLSNHM 01942 represents a juvenile *Jeholosaurus*. The endosseous labyrinth is tentatively reconstructed, although the disarticulation of the neurocranial bones hampers its complete reconstruction. Thanks to the analysis of previously undescribed inner neurocranial bones (such as the prootics, the exoccipital/ophistotic, basioccipital, and basisphenoid), we improve the previous phylogenetical scoring for *J*. *shangyuanensis*, and perform a phylogenetical analysis adding the basal ornithopod *Changmiania liaoningensis* and the recently re-evaluated *Ajkaceratops kozmai*. The phylogenetical analysis reports a well-supported base of Ornithopoda, with *C*. *liaoningensis* as the most basal ornithopod, and a resolved topology for *Nanosaurus agilis*, *Changchunsaurus parvus*, *Haya griva*, *Yandusaurus hongheensis* and *J*. *shangyuanensis*.

## Introduction

*Jeholosaurus shangyuanensis* is a small-bodied ornithischian from the Early Cretaceous Yixian Formation of Liaoning province in China, initially described by Xu et al. [1] based on two nearly complete skulls and partial postcranial material and recovered as Ornithopoda *incertae sedis*. The phylogenetic history of the species is complex, especially due to the instability of the basal portion of the Ornithopoda tree. After Xu et al. [1] suggested that *Jeholosaurus* could form a clade with *Agilisaurus*, *Hexinlusaurus* and *Xiaosaurus* without a phylogenetic analysis to support this claim, Butler et al. [2] recovered *Jeholosaurus* as a basal member of Ornithopoda in an unresolved polytomy with *Hypsilophodon*. The study was based solely on the two individuals known at that time, the holotype IVPP V 12529, a dorsoventrally compressed skull with neurocranial elements visible in ventral view, and IVPP V 12530, a referred skull embedded in matrix with the lateral side well exposed without distortion, but with inner skull bones hidden by the matrix. Barrett and Han [3] added new information on the osteology of the skull and mandible thanks to new materials (IVPP V 15716, IVPP V 15717, IVPP V 15718 and IVPP V 15719) from the same locality and horizon as the holotype. The authors confirmed the position of the taxon within Ornithopoda, but with *Hypsilophodon* found to be closer to iguanodontians than *Jeholosaurus* [3]. Furthermore, a “Chinese clade” with *Agilisaurus* and *Hexinlusaurus*, as proposed by Xu et al. [1], was not supported as these taxa were found to lie outside Cerapoda. Makovicky et al. [4] subsequently described the Mongolian taxon *Haya griva*, a neornithischian sharing a bifid caudal ramus with *Jeholosaurus*. This single, unambiguous synapomorphy unite a small ornithopod Asian clade, formed by *Jeholosaurus*, *Haya* and *Changchunsaurus* (although the bifid caudal ramus is not found in the latter species), located near the base of the ornithopod radiation as more derived than *Orodromeus* but less derived than *Hypsilophodon* [4]. The poorly resolved phylogenetic position of *Jeholosaurus* was hampered by the lack of decent postcranial material, a situation solved later by Han et al. [5]. The authors described postcranial material associated to the known skulls plus a new, undescribed skeleton (IVPP V15939). The phylogenetic resulted in a strict reduced consensus tree that placed *Jeholosaurus*, *Changchunsaurus* and *Haya* together within a clade of basal ornithopods named Jeholosauridae, with *Jeholosaurus* as sister taxon of *Changchunsaurus* [5]; it was also hypothesized that *Koreanosaurus* and *Yueosaurus* might belong to this clade as well. However, those analyses showed how the base of Ornithopoda remains mostly unresolved. In a large revision of neornithischian phylogeny, Boyd [6]incorporated data from published and unpublished *Jeholosaurus* specimens, recovering a clade consisting of *Jeholosaurus* and *Yueosaurus* supported by numerous synapomorphies (distinct “trench” between the greater trochanter and the head of the femur; 6+ sacral vertebrae; lateral swelling of the ischiadic peduncle of the ilium; lesser trochanter of the femur that is anteroposteriorly narrow and closely appressed to the greater trochanter with its dorsal extent approximately level with the head of the femur). In Boyd’s [6] analysis, *Changchunsaurus* and *Haya* are recovered within Thescelosaurinae, different from the previously proposed Asian clade of Jeholosauridae. In the description of the new Australian ornithopods *Galleonosaurus* and *Qantassaurus*, Herne et al. [7] retrieved the Jeholosauridae (*Haya*, *Jeholosaurus*,and *Changchunsaurus*), but outside Ornithopoda and as sister group to Thescelosauridae. Lastly, Dieudonnè et al. [8] relocated *Jeholosaurus* as a basal member to Ornithopoda but without forming a single clade with *Changchunsaurus* and *Haya*.

Here we describe a new specimen of *Jeholosaurus shangyuanensis*, YLSNHM 01942, consisting of an articulated skull but lacking its postcranial skeleton. The examination is done on visual observations plus the use of computed microtomography (μCT) technology to reconstruct and visualize inner bones of the neurocranium, providing a series of osteological features previously hidden and/or unknown from the previously published specimens.

## Material and methods

**Fig 1:**
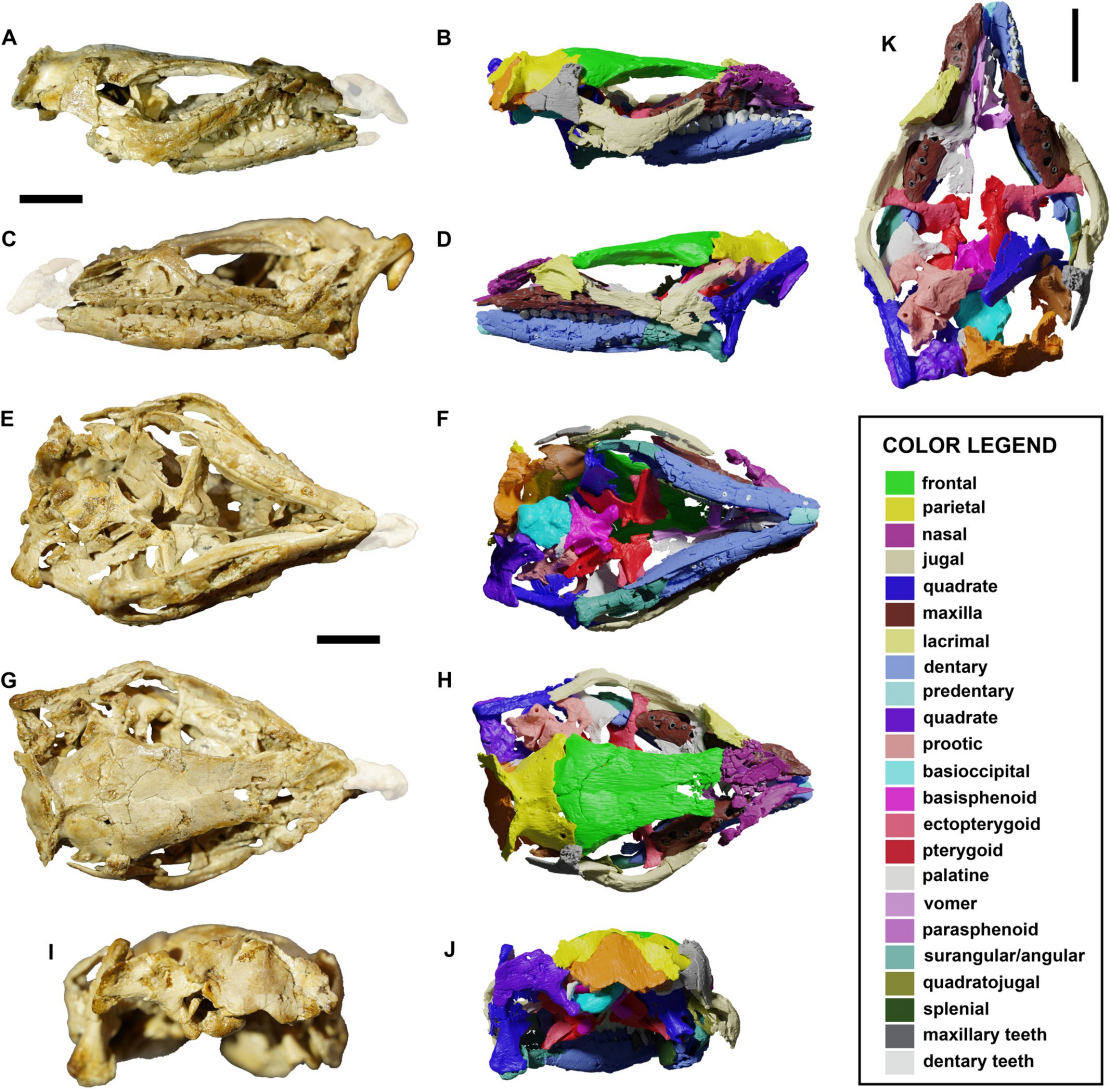
The skull YLSNHM 01942 of *Jeholosaurus shangyuanensis* and segmented model of the same in right lateral (A-B), left lateral (C-D), ventral (E-F), dorsal (G-H) and caudal (I-J) views. Dorsal view of the skull without the frontal, parietal and nasal to show the innermost cranial bones (K). The blurred regions correspond to artificially added bone fragments, not belonging to the specimen. Scale bars = 1 cm.

The skull described in the present paper was collected in the Lujiatun Beds close to Lujiatun Village. This specimen was only partially prepared when it was acquired by the YLSNHM; it was subsequently carefully prepared by one of us (NV-G). During preparation and subsequent digitization, we determined that, while the specimen is genuine, the two bones located rostrally and the anterior part of the predentary were artificially added during early restoration of the specimen (visible in Fig 1), and we removed them. Pictures were taken with a Sony A7c (f/5.6, ISO 100, 35 mm focal length) and the measurements with a manual caliper. The osteology of YLSNHM 01942 was analyzed macroscopically using a stereoscope and following anatomical descriptions by Barrett and Han [3], Herne et al. [7] and Barta and Norell [9]. To visualize and describe the inner aspects of the skull bones, scanning was completed using a μCT RX EasyTom (http://www.rxsolutions.fr). Images were generated at a voltage of 105 kV and a current of 285 μA. This generated 4353 images and a voxel size of 0.0223509 mm. Segmentation was performed using Avizo 3D 2021.1. Reconstruction, visualization and analysis were performed using Blender 2.93. Each bone was imported in the software, cleaned from particles and disassociated pixel groups, smoothed (factor 1, repeat 8), and finally rendered. Orthogonal pictures were taken for each bone following standard direction using Eevee rendering, whereas Cyclic rendering was used for the articulated skull. Finally, a hypothetical re-assembled skull was assembled following articular surfaces of the bones. 3D mesh files are available on figshare at the following DOI: 10.6084/m9.figshare.26065342, the full μCT data can be consulted upon request. The study was authorized by YLNSHM Board of Directors, and no permits were required for the described study, which complied with all relevant regulations.

Even though *Jeholosaurus* was already scored in different matrices [5, 6, 8, 10, 11], the excellent preservation quality of YLSNHM 01942 allows to score characters previously unknown in the species. We review previous scorings for the species, updating the phylogenetical matrix from Rotatori et al. [11] (based on Dieudonnè et al. [8], containing SHN.015.JJS.LPP, *Hesperonyx martinhotomasorum* and *Draconyx loureiroi* and maintaining the taxonomic assumptions of Rotatori et al. [11]). We added the fragmentary rhabdodontid *Transylvanosaurus platycephalus* [12] and *Changmiania liaoningensis*, a basal ornithopod from the Lower Cretaceous of the western Liaoning Province in China [13], taxa not present in Rotatori et al. [11], and the recently re-evaluated *Ajkaceratops kozmai* [14], considering it as a basal ceratopsian by using the first OTU provided by the authors. *Psittacosaurus mongoliensis* and *Yinlong* are also updated according to Czepiński and Madzia [14]. Character 59 and 73 in Dieudonnè et al. [8] were found to be describing the same features, therefore we deleted ch.59. Then, we solved characters previously scored as ‘?’ in *Jeholosaurus*: 31, 46, 78, 82, 83, 102, 106, 107, 108, 109, 111, 114, 116, 119, 120, 127, 146, 149, 167, 170, 171, 176, 179, 182, 186, 187, 189, 190, 191, 192. The final dataset contains 78 taxa and 342 characters (with the first character considered as null following Dieudonnè et al. [8]), it was modified in Mesquite and run using TNT V. 1.5 (Tree Analysis using New Technology, [15]). We treated the same characters as ordered in Dieudonnè et al. [8]: Ch.110, Ch.150, Ch. 203, and Ch. 282. We used the parsimony script provided by Rotatori et al. [11], expressed also by the TNT command *drift*: *fitdiff 5 rfitdiff 0*.*1; sectsch*: *rss minsize 5; sectsch*: *css minfork 5 rounds 100; xmult*: *rss css drift 100 ratchet 100 fuse 10 replications 10 hits 10;*. We followed the same methodology for the new technology search strategies, as well as the equal weighting (EW) and extended implied weighting with K = 15. The final trees were finally edited in Adobe Illustrator.

## Results

The *Jeholosaurus* skull YLSNHM 01942 is small, only 5.9 cm in length, measured from the rostralmost point of the nasal to the caudalmost margin of the occiput. It suffered from strong diagenetic deformation, as it is dorsoventrally flattened and mediolaterally expanded in cranial view, making the left orbit wider than the right one (Fig 1). The right quadrate and prootic are taphonomically pushed towards the left side, so that the right prootic is located ventrally to the right one. The maximum width is 3.2 cm, calculated from the flexion point of the jugals. The external narial fenestrae, greatly expanded in other *Jeholosaurus* skulls [3], are not present, due to diagenetical breakage and distortion of the nasal-premaxilla complex, as well as the antorbital and infratemporal fenestrae. The orbits are suboval in shape, dorsoventrally compressed and rostrocaudally elongated, corresponding to 40% of the total skull length. The left orbit is 1.8 cm wide, contra 0.8 cm for the right orbit. The premaxillae are missing, and the elongated element located on the anterior right side is tentatively referred to the left nasal (Fig 1). The palpebrals are not preserved. Some neurocranial elements moved towards the left side, producing a strong asymmetry in the skull. Four bones were segmented along with the others, but we were not able to properly identify them (S1 Fig).

### Maxilla

**Fig 2:**
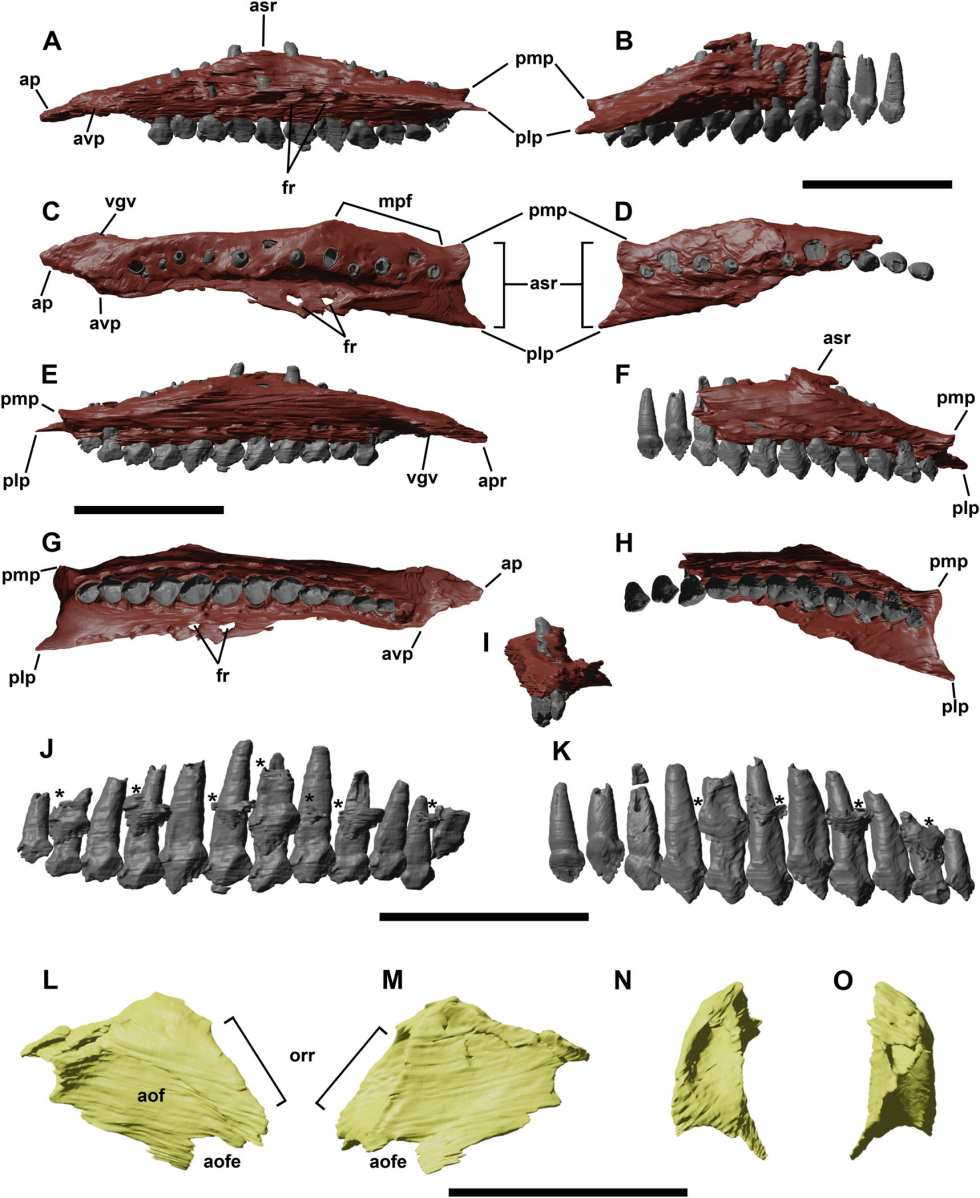
Left and right maxillae of YLSNHM 01942 in left (A,B), dorsal (C,D), medial (E,F), ventral (G,H) and rostral (I) views. Left maxillary (J) and right maxillary (K) teeth in lingual views, the asterisks indicate replacement teeth. Lacrimal of YLSNHM 01942 in left (L), medial (M), caudal (N) and rostral (O) views. Abbreviations: **aof**, antorbital fossa; **aofe**, antorbital fenestra; **asr**, ascending ramus of the maxilla; **ap**, anterior (premaxillary) process; **avp**, anteroventral process; **fr**, foramina; **mpf**, medial palatine facet; **orr**, orbital rim; **plp**, posterolateral process; **pmp**, posteromedial process. Scale bars equal 1 cm.

The maxillae of YLSNHM 01942 are dorsoventrally compressed and mediolaterally curved (Fig 2A–2H); teeth are preserved on both elements and the general morphology can be analyzed on the left element (Fig 2J and 2K). The taphonomical compression provoked the roots of the teeth to be exposed on the dorsal surface of the maxillae. The maxilla is an elongated, subtriangular bone, bordered by the jugal caudally, the lacrimal dorsorostrally, the vomer mediorostrally and the ectopterygoid mediocaudally. The articular contact with the premaxilla is not preserved. The maxillary teeth are present along the horizontal tooth row that is inset to form a deep buccal emargination as in other *Jeholosaurus* [3], bordered dorsally by a horizontal ridge. This ridge extends rostrally from the ventral border of the jugal to the ventrocaudal margin of the antorbital fossa. Between the tooth row and the ridge, four nutrient foramina open on the lateral side of the maxilla. Rostrally, the maxillary emargination is curved medially to form the ventral margin of the antorbital fenestra, but the rising, hook-like ascending process is missing. However, the rostral margin of the lacrimal bears the articular surface with this process, showing a crescentic shape with a concave side facing medioventrally. A small and slightly displaced antorbital fenestra is visible laterally, between the ventral side of the lacrimal and the medial side of the maxilla, and the fenestra is in continuation with a small opening visible in dorsal view. Here, a gentle slope extends medioventrally from the articular point between the jugal and the maxilla along a narrow channel that corresponds to the antorbital fenestra. On the medial shelf of the maxilla, the tooth roots pierce through the bone so that the tip of their root exits prominently from the outline of the bone. This is a condition shared by all maxillary teeth in both the left and right elements, but it remains unclear whether it can be explained by the dorso-ventral post-mortem compression of the bone or it is a pathological character. A short edentulous diastema is preserved in YLSNHM 01942, equivalent to the mesiodistal length of 1 to 2 crowns, as in *Changchunsaurus* [16] and *Haya* [4].

### Lacrimal

Only the left lacrimal is preserved in YLSNHM 01942 (Fig 2L–2O) and, even though this bone was already described partly by Barrett and Han [3], the segmented model offers the possibility to describe its medial and caudal sides. The lacrimal has a curved outline in lateral view, pointing rostrally. Its dorsal surface is flat to receive the prefrontal and the nasal, whereas its caudoventral margin forms the rostroventral corner of the orbital rim. The medial side of the lacrimal expands into a sub-squared flat sheet of bone, representing the caudal part of the inner wall of the antorbital fossa. In cranial view, the medial side is strongly curved, whereas the lateral side of the main body of the lacrimal is flatter. The caudal surface has a rostrocaudally elongated triangular shape, wider at the base and tapering to a point on the dorsal side. It is slightly concave, and a large lacrimal duct enters the dorsal part of the bone, opening rostrally in the contact surface with the nasal.

### Nasal

**Fig 3:**
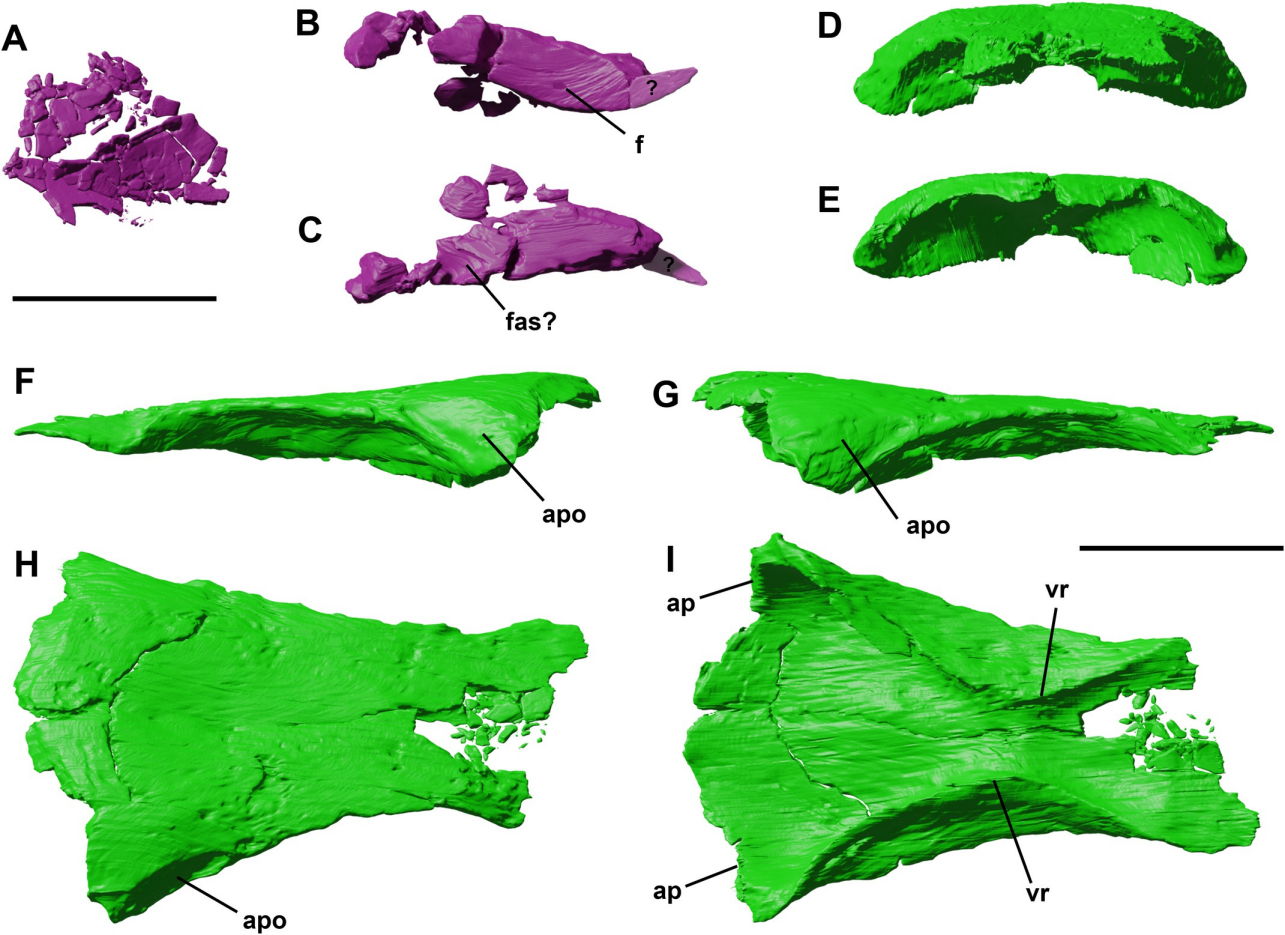
Fragmentary and poorly preserved left nasal of YLSNHM 01942 in dorsal view (A). Possible right nasal in lateral (B) and dorsal views (C) Frontal complex of YLSNHM 01942 in rostral (D), caudal (E), left lateral (F), right lateral (G), dorsal (H) and ventral (I) views. Abbreviations: **apo**, articular surface for postorbital; **ap**, articulation surface for parietal; **fas**, frontal articular surface; **f**, foramen; **vr,** ventral ridge. Scale bars equal 1 cm.

The left nasal is preserved rostrally to the frontal. However, this element is strongly taphonomically dismembered, and neither its original shape nor any osteological features can be confidently recognized and adequately described (Fig 3A). However, the long fragment on the right side of the skull is here tentatively referred to the nasal because of the presence of a lateral foramen (Fig 3A). In fact, *Jeholosaurus* shows a series of foramina on the nasal, considered an autapomorphy by Barrett and Han [3]. The lateral and medial surfaces are flat and rostrocaudally elongated, slightly diverging rostrally to each other so that the rostral section is wider than the caudal one. On the caudal end, a U-like depression might correspond to the articular surface of the frontal (Fig 3B).

### Frontal

Both frontals are preserved, although their rostral end is fragmented and the original shape and articulation with the nasals and prefrontal are lost (Fig 3B–3G). The straight midline contact between the two frontals is still visible, suggesting the two elements were not yet fused at the moment of death, as reported for the holotype and one referred specimen by Barrett and Han [3]. Considering a unique bone formed by the two partial frontals, the frontal is a long bone forming most of the entirety of the skull roof. It narrows rostrally and thickens caudally. It suffers from taphonomical compression, but its original shape is not compromised, by comparison with the other specimens described by Barrett and Han [3]. A shallow concavity is located towards the middle region of the dorsal surface. The ratio between its maximum length (measured from the caudalmost central point in articulation with the parietal) and its widest point (measured between the postorbital articular facets) is 2.7, close to the value measured in the holotype and other early ornithopods [3] (Barrett and Han, 2009), and lower than non-iguanodontian ornithopods and ceratopsians [3] (Table 1).

**Table 1:** Length/width ratio of the frontal bones in different neornithischians, modified from Barrett and Han [3] (2009).

Genus	Ratio	References
*Jeholosaurus* YLSNHM 01942	2.7	This study
*Changmiania*	>4	[13]
*Jeholosaurus*	3.0	[3]
*Agilisaurus*	3.0	[3, 17]
*Hypsilophodon*	3.2	[3, 18]
*Zephyrosaurus*	3.0	[3, 19]
*Liaoceratops*	2.2	[3, 20]
*Orodromeus*	2.2	[3, Scheetz, R.D. [Unpublished]]
*Psittacosaurus*	1.8	[3, 21]
*Thescelosaurus*	1.9	[3, 22]
*Yinlong*	1.8	[3, 23]

The contact line with the parietal has an enlarged U-shape, and the left lateralmost corner between the frontal and the parietal is separated by a deep straight groove, not present on the opposite side. The orbit rim is sharp and thin, and it forms approximately 50% of the orbital fenestra as reported by Yang et al. (2020). On the caudolateral surface, the articular facets for the rostral process of the prefrontals are both visible on both sides. They are elongated sulci, tapering rostrally. In lateral view, part of the ventral surface is visible from the orbit, flattened and curved medially with a prominent medial ridge.

In ventral view, two symmetrical low rounded ridges, the *cristae cranii* [*sensu* 24] (Fig 3G) form the medial rim of the orbital cavity and extend to the medial surface of the postorbital. Caudally, these ridges border the region of the cerebral hemisphere, whilst rostrally they encapsulate the olfactory tract, which is wider than in *Lesothosaurus*. Between these two regions, at the point of minimum separation between the two *cristae cranii*, there is a transversal blunt ridge, likely corresponding to the ventral cerebral inflection between the olfactory bulb and the cerebral hemispheres.

### Postorbital

**Fig 4:**
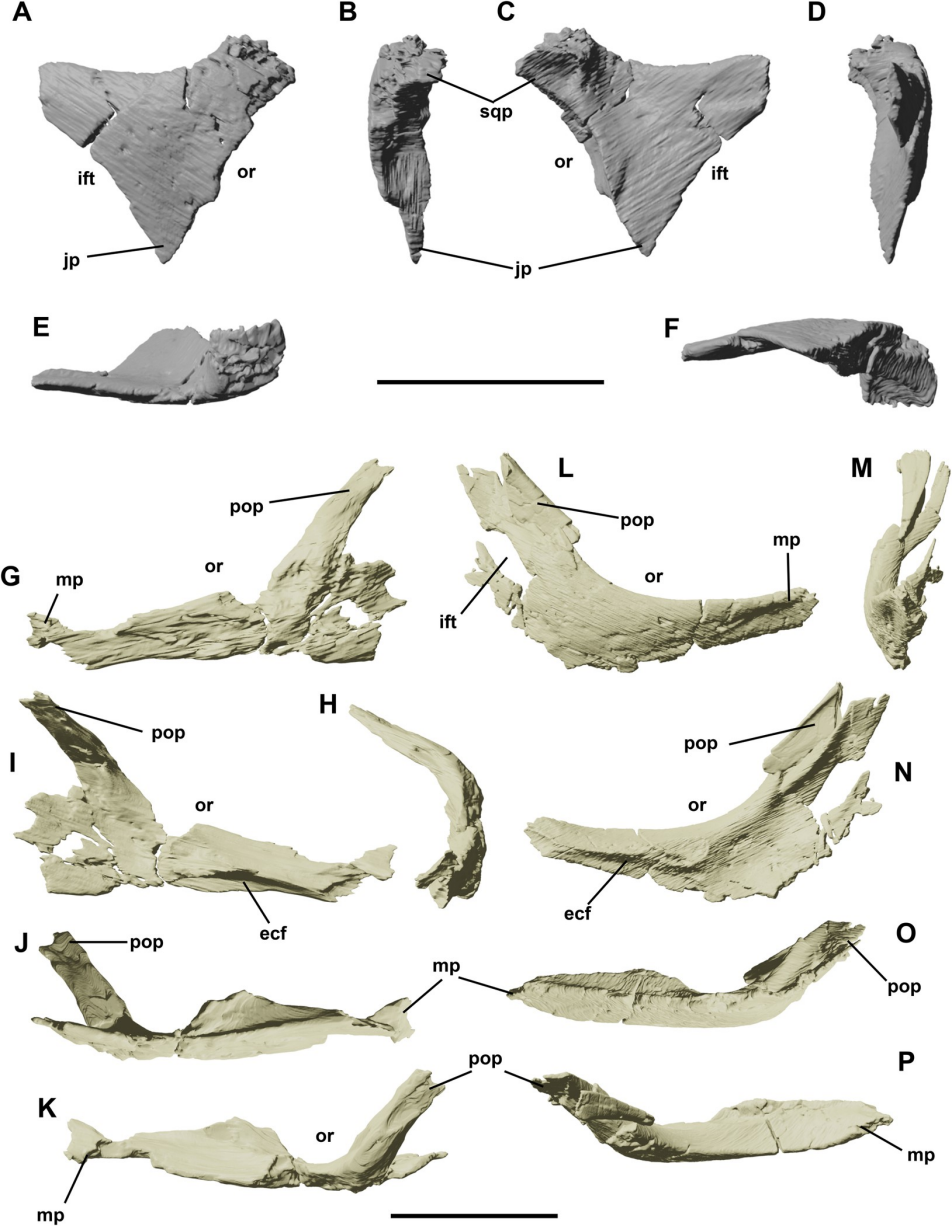
Right postorbital of YLSNHM 01942 in lateral (A), rostral (B), medial (C), caudal (D), dorsal (E) and ventral (F) views. Left and right jugals of YLSNHM 01942 in lateral (G,L), medial (I,N), rostral (H,M), ventral (J,O) and dorsal (K,P) views. Abbreviations: **ecf**, ectopterygoidal facet; **jp**, jugal process; **ift**, infratemporal fenestra; **mp**, maxillary process; **or**, orbit; **pop**, postorbital process; **sqp**, squamosal process. Scale bars equal 1 cm.

Only the left postorbital is preserved in YLSNHM 01942 (Fig 4A–4F). This triradiate element is not fused to the frontal, and it is caudally shifted from its original position, revealing the articular surface with the caudolateral margin of the frontal. The breath between the rostral and the caudal process is 10.8 mm, whilst the total length (measured from the caudal and the distalmost point of the ventral process) is 14.3 mm. The rostral process is incomplete and truncated, missing the putative ornamented rostral end that articulated with the frontal. It is the thicker process of the postorbital, expanding mediolaterally and curving abruptly in dorsal view at about 90 degrees to contact the frontal. Just ventral to this process, a small and smooth recess indicates the articular surface for the supraorbital or accessory supraorbital, as in *Iguanodon* [25] and *Thescelosaurus* [26]. The ventral process is broken off and medially offset from the main axis of the postorbital. Towards the contact with the jugal, it tapers with a triangular cross-section. The caudal process is the mediolaterally thinner, gently curved medially in dorsal view, and straight (not twisted as in *Thescelosaurus* [26]). The distal end shows a central inflation that suggests a faint bifurcation, not as developed as in *Thescelosaurus* but seen in other *Jeholosaurus* specimens [3] and in *Haya* [4].

### Jugal

Both jugals are present, although at different levels of preservation (Fig 4G–4P). The jugal forms the ventral border of the orbital fenestra and part of the infratemporal fenestra, but does not participate in the small antorbital fenestra, as already reported by Barrett and Han [3]. It is a triradiate bone, although its morphology seems different in the left and the right elements. Rostrally, the maxillary process is the longest; its ventral and dorsal borders are subparallel along the main shaft, but they taper to a point close to the articulation with the lacrimal. This contact is not preserved in either the left or right element. In dorsal view, the jugal is strongly bowed, as in *Changchunsaurus* [16], *Haya* [4] and *Changmiania* [13]. The lateral side of the jugals lacks the diagnostic ornamentation of the species, but Barrett and Han [3] and Jin et al. [16] suggested this might be related to ontogeny, a suggestion corroborated by the apparent immaturity of the specimen, based on degree of fusion of skull elements. The jugal also lacks the boss-like structure, located at the junction between the dorsal and rostral rami in *Haya* [4]. The medial side of the maxillary process forms a medial thickened cleft for articulation with the maxilla and ectopterygoid, as in other basal ornithopods and neornithischia (*Haya*), different from the vertical facet in *Probactrosaurus* [27] and *Protohadros* [28]. The dorsal ramus of the jugal, or postorbital process, is preserved on the left jugal: it forms the caudoventral border of the orbital margin and the rostroventral border of the infratemporal fenestra. In cross-section, it has a crescent shape with the concavity facing medially. Laterally, this process has an elongated V-like articular surface for the ventral process of the postorbital. The caudal ramus, or quadratojugal process, is visible on the right jugal. It is elongated as in IVPP V15716 [3], and it forms the caudoventral margin of the infratemporal fenestra. The left jugal does preserve a poorly-preserved section of the caudal ramus. Unlike the right one, there is a small U-like margin, suggesting a bifurcation of this process.

### Squamosal

**Fig 5:**
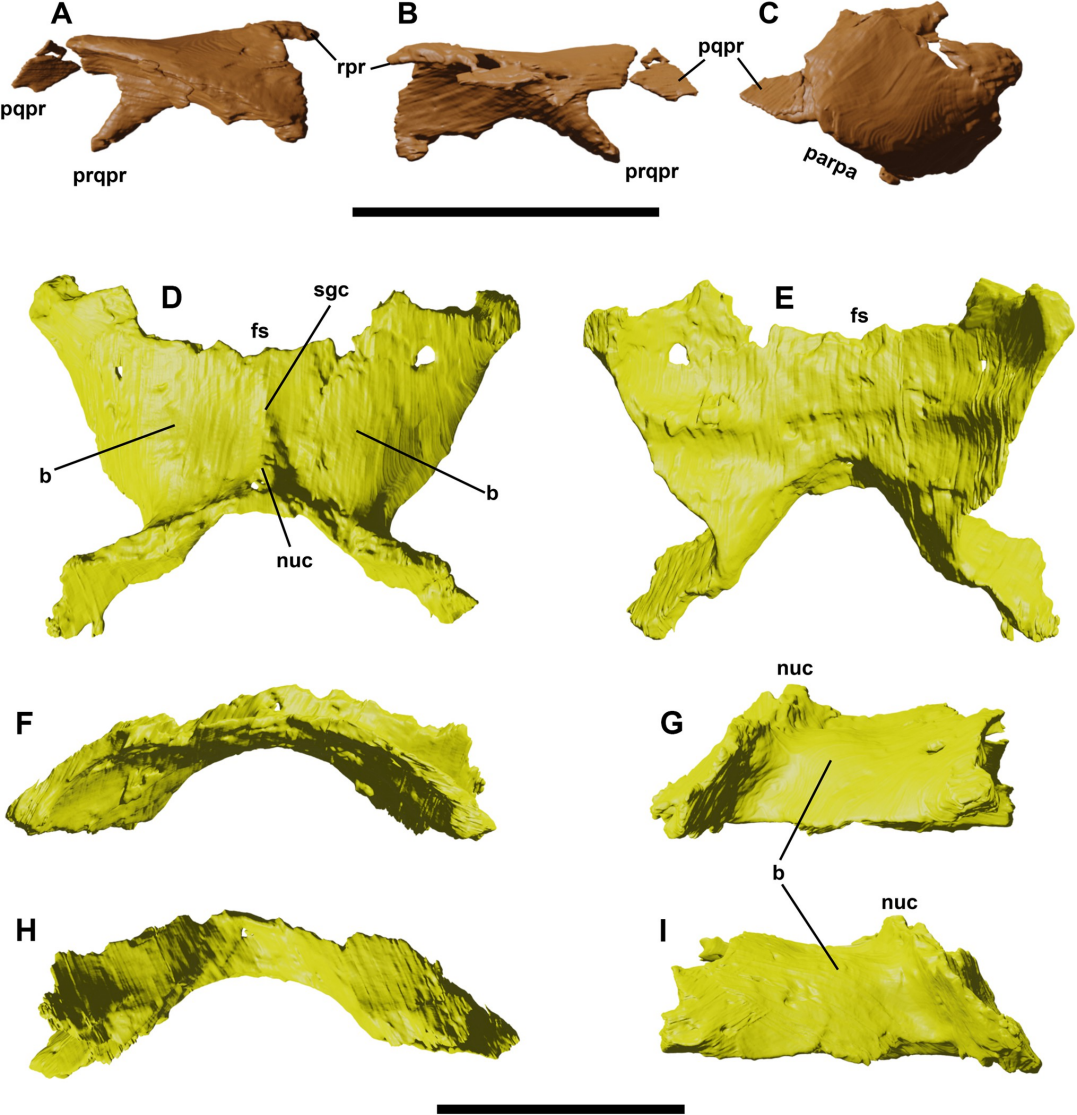
Right squamosal of YLSNHM 01942 in medial (A), lateral (B) and dorsal (C) views. Parietals of YLSNHM 01942 in dorsal (D), ventral (E), rostral (F), right lateral (G), caudal (H), and left lateral (I) views. Abbreviations: **b**, bump; **fs**, frontal surface; **nuc**, nuchal crest; **parpa**, articular surface for the paroccipital process; **pqpr**, postquadratic process; **prqpr**, prequadratic process; **rpr**, rostral process; **sgc**, sagittal crest. Scale bars equal 1 cm.

The right squamosal squamosal is preserved in YLSNHM 01942 but is displaced between the postorbital and the quadrate (Fig 5A–5C). In lateral view, its rostral process is eroded; in rostral view, it is dorsoventrally thin and regularly curved, so it likely covered the caudal process of the postorbital. The dorsal surface of the squamosal is flat and inclined mediocaudally. A strong and rounded dorsal ridge extends from the ventral side of the rostral process up to the rostral border of the postquadratic process, so that the prequadratic process is strongly inset in lateral view. In front of the prequadratic process, this depressed area served as attachment site for the adductor musculature (*M*. *adductor mandibulae superfcialis*) [16, 18]. The prequadratic process is dorsoventrally high and triangular in lateral view and in cross-section. It is unusually inclined caudally, but this is likely due to postmortem deformation. A ventral ridge extends between the posterior margin of the prequadratic process and the anterior margin of the postquadratic process, enclosing the medial surface of the socket for the head of the quadrate. The postquadratic process is shorter than the prequadratic one and mediolaterally compressed. Its lateral side flares caudodorsally. The medial process of the squamosal is a stout sheet of bone that extends rostromedially from the caudomedial margin of the postquadratic process. In caudal view, the ventral margin of the squamosal is regularly concave and fitted against the dorsal border of the paroccipital process.

### Parietal

The parietals in YLSNHM 01942 are fully fused, as in the holotype and the referred materials in Barrett and Han [3] (Fig 5G–5L). A low but prominent sagittal crest extends along the midline to contact the two frontals rostrally and the separation points of the two caudolateral processes of the parietal. The contact with the frontals is preserved, especially the left one. Here, the surface of the parietal is crushed by the plastic deformation of the skull, showing the articular contact running at 60 degrees from the sagittal crest. Near the postorbital corner, the contact surface between the parietal and the frontal turns abruptly, forming a deep sulcus extending towards the postorbital articular surface. Two faint bumps are visible rostrally to the caudolateral processes, a trait not visible in other *Jeholosaurus* skulls. Different from these materials is also the overall shape of the dorsal surface of the parietal. In fact, in YLSNHM 01942 the paired parietals are laterally widened, with a less marked constriction described for the other skulls by Barrett and Han [3], and they turn ventrally to form the dorsal sidewall of the braincase. This odd morphology might be related to the strong dorsoventral compression of the skull, but the symmetry of the two parietals suggests otherwise.

The rostrolateral corner of each parietal is flat, receiving the dorsomedial portion of the postorbital; the rostrocaudally expanded process reported by Barrett and Han [3] is missing. The caudolateral processes are long and narrow, and they form the articular surface for the supraoccipital. These two bones are not fully fused between each other, as suggested by the presence of an opening on the dorsal contact between the supraoccipital and the mid-point of the paired parietals. The shape of the supratemporal fenestra cannot be adequately described because of the absence of complete squamosals and postorbitals; however, the greatest part of its margin was apparently formed by the parietal.

### Quadrate

**Fig 6:**
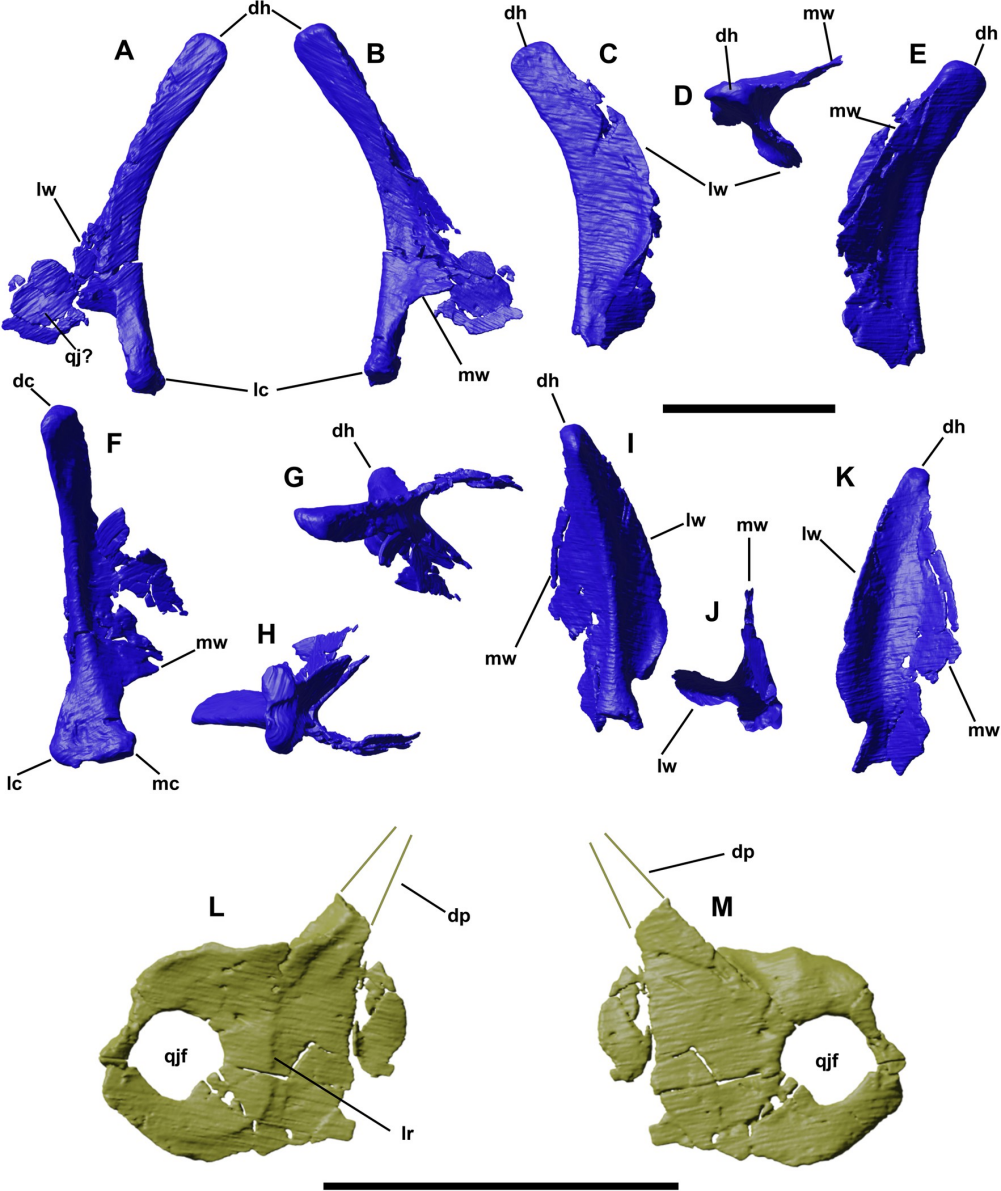
Left and right quadrates of YLSNHM 01942 in lateral (A,C), medial (B, E), caudal (F,I), dorsal (G,D), ventral (H,J), and rostral (K) views. Left quadratojugal of YLSNHM 01942 in lateral (L) and medial (M) views. Abbreviations: **dh**, dorsal head; **dp**, dorsal process; **lc**, lateral condyle; **lr**, lateral ridge; **lw**, lateral wing; **mw**, medial wing; **mc**, medial condyle; **qj**, quadratojugal; **qjf**, quadratojugal foramen; **vc**, ventral condyle. Scale bars equal 1 cm.

Both quadrates are preserved in YLSNHM 01942; the right one is strongly dislocated within the endocranium, whilst the left one is deformed and slightly crushed (Fig 6A–6K). The quadrate is a long and narrow pillar bone, connecting the squamosal dorsally and the surangular ventrally. In lateral view, the left quadrate has a strong curvature on the upper two/third of its shaft, so that its proximal end is located caudal to its distal end. However, the right quadrate exhibits a weaker curvature, suggesting that the right element suffered from taphonomical deformation. The proximal end, corresponding to the articulation with the squamosal, is rounded in lateral view and triangular in dorsal view. A strong ridge extends from the caudoventral side of its proximal end along its main shaft, fainting next to its distal end. Two wide flanges originate from the shaft of the quadrate: the lateral quadratojugal wing projects rostrally to contact the quadratojugal (although the exact nature of the contact is missing), and the medial pterygoid wing projects rostromedially, covering the quadratic ramus of the pterygoid. The lateral surface of the quadratojugal wing is flat, different, although it is usually excavated by a shallow sulcus in other basal ornithopods [4, 29] and it has been previously reported as concave in *Jeholosaurus* [3]. On the posteromedial side of the quadrate wing, just dorsal to the condyles, there is a shallow fossa, reported also in IVPP V12529 [3] as well as in *Parksosaurus*, *Orodromeus*, *Zephyrosaurus* and *Thescelosaurus* [19, 26, 30]. The distal end is truncated in the right quadrate, showing a crescentic cross-section with the concavity facing rostrally. The left quadrate is in articulation with the surangular, showing the morphology of its lateral and medial distal condyles. Above the former, a small triangular bump is developed caudolaterally. The two condyles are not clearly separated, with just a faint and short groove between them. The lateral condyle is roundish, whilst the medial looks flatter and located slightly more ventrally than the lateral one, as reported in *Jeholosaurus* [3].

### Quadratojugal

Only the right quadratojugal has been detected thanks to the segmentation (Fig 6L and 6M), even though a fragment associated with the left quadrate might represent the left quadratojugal (Fig 6A). The right element is a broad and transversely flattened bone, like in the holotype and referred materials of *Jeholosaurus*, as well as in *Haya* and *Hypsilophodon*. Its main body is sub-squared, different from the elongated and slender one visible in IVPP V15716. Barrett and Han [3] describe the rostral portion of the quadratojugal tapering to a point, but this morphology is not recognized in YLSNHM 01942. Furthermore, the “ventrolateral tab-like process” [*sensu* 3] is not present either, and the smooth and contiguous surface in this area suggest it is not a taphonomical artifact. The quadratojugal foramen is located caudally and not centrally as reported by Barret and Han [3]; it is large, almost occupying half of the entire area of the main body. A distinct ridge extends perpendicularly in the middle of the bone. The area rostral to the lateral ridge might correspond to the articular surface for the jugal. The dorsal process is missing, truncated at the base of the proximal portion. This process has a sub-triangular and narrow cross-section. The dorsal process diverges from the main body with an angle of 135 degrees, in contrast with the almost vertically directed process in the holotype IVPP V12530.

### Palatine

**Fig 7:**
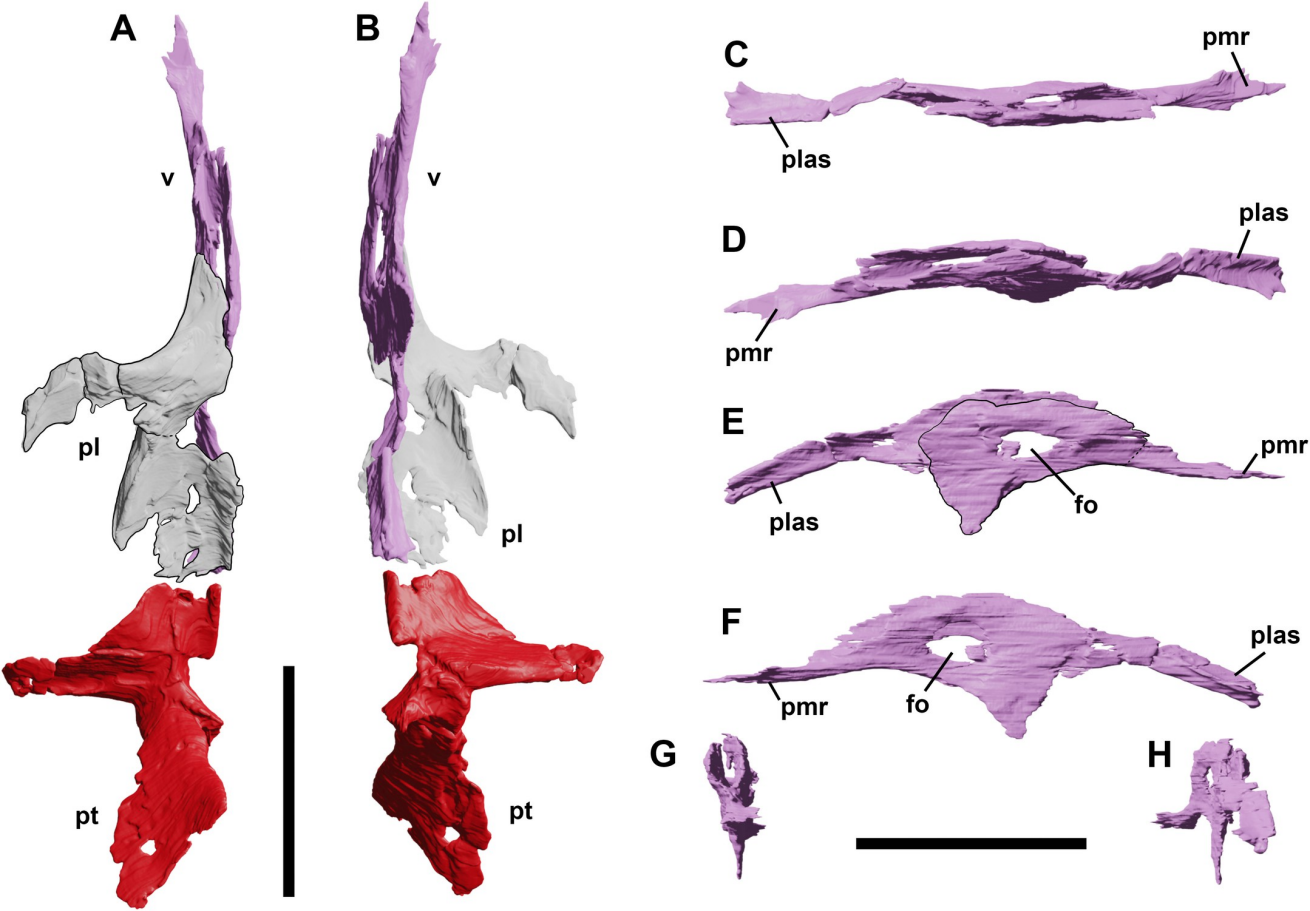
Palatal complex of YLSNHM 01942 in dorsal (A) and ventral (B) views. Vomers of YLSNHM 01942 in dorsal (C), ventral (D), left lateral (E), right lateral (F), rostral (G) and caudal (H) views. Abbreviations: **fo**, foramen; **pl**, palatine; **plas**, palatine articular surface; **pmr**, premaxilla ramus; **pt**, pterygoid; **v, comer**. Scale bars equal 1 cm.

The left palatine is preserved in connection with the other palatal elements (Fig 7A and 7B). Its rostral part forms a mediolaterally compressed process, roughly triangular in dorsal view, with a dorsoventrally concave medial surface that covers the caudolateral side of the vomer. The lateral side of the rostral process progressively raises dorsolaterally to form a broad dorsal process. This process is deformed by the dorsoventral compression of the skull, lying against the rostromedial side of the maxilla; it had a more vertical orientation in life, so that it participated in the medioventral wall of the orbit. The thinner caudal portion of the dorsal process is only partly preserved, so that its precise shape cannot be adequately reconstructed. The caudal portion of the palate forms a prominent maxillary process, triangular in dorsal view and in cross-section. The height of its medial side progressively increases cranially so that it forms a dorsoventrally concave and rostroventrally straight contact surface with the rostromedial side of the dentary. The maxillary process extends laterally as a thin sheet of bone to contact with the pterygoid.

### Vomer

Medially to the maxilla, the elongated vomers extend rostrocaudally to insert in the maxillae and contact the pterygoids, lying on the palatine ventrolaterally (Fig 7A and 7B). The left vomer is more complete than the right one, the latter preserving only the main, central portion with a large foramen. The vomer is a thin, sheet-like bone, divided into three parts: the caudal ramus, the central body and the rostral ramus (Fig 7C–7H). The caudal ramus contacts the medioventral side of the palatine and is triangular in cross-section. The central body is formed by the left and right vomers, pierced centrally by a paired large foramen. The medial side of both vomers are separated, forming the ala of the vomer, facing each other and forming an internal deep, scoop-like vomerorostral canal. The two vomers fuse into an extensive and pointed triangular ventral process, similar to the condition reported by Galton [31] in *Hypsilophodon*, although in this process is more roundish and less pointed in the latter. The rostral process is dorsoventrally flattened, tapering to a point.

### Pterygoid

**Fig 8:**
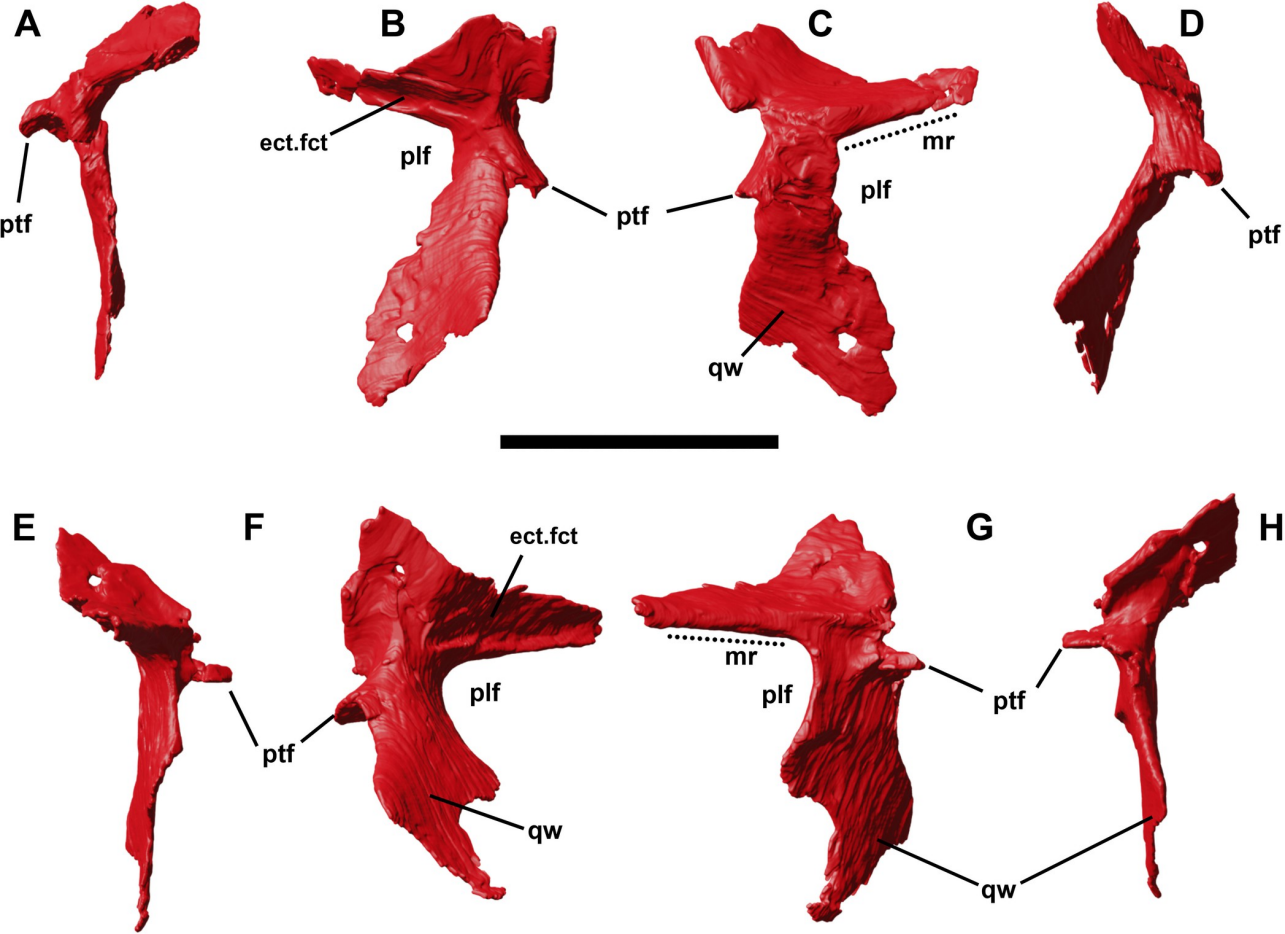
Left and right pterygoids of YLSNHM 01942 in lateral (A,E), dorsal (B,C), ventral (F,G) and medial (D,H) views. Abbreviations: **ect.fct**., ectopteryoidal facet; **mr**, mandibular ramus; **plf**, palatal fenestra; **ptf**, pterygoidal flange; **qw**, quadrate wing. Scale bar equals 1 cm.

Both pterygoids are preserved in YLSNHM 01942, although they present a certain degree of taphonomical deformation (Fig 7A and 7B). It is a triradiate, dorsoventrally thin element constituted by a rostral palatal ramus, a transverse flange and a quadrate ramus (Fig 8A–8H). The central body is thicker than the rami, and it is slightly arched in medial view, with the palatal ramus upturning dorsocaudally. It is short and subtriangular, with a bony rod on its medial side that might represent a detached fragment from the palatine or the vomer. The rostral ramus is in continuation with the transverse flange, forming a subtriangular rostral portion of the pterygoid. A longitudinal ridge along the entire length of the transverse flange identifies the articular facet for the medial, axe-shaped side of the ectopterygoid. In contrast with IVPP V12529 and IVPP V15718 [3], the transverse flange does not get thicker dorsoventrally toward the distal end. The main central body of the pterygoid bears a medial, curved process that likely articulated with the paired rostral process of the basisphenoid. The quadrate ramus corresponds to the largest portion of the bone. It is a very thin subtriangular process, tapering to a point towards its caudalmost end. Given the asymmetrical shape and the fragments close to the caudal end, it is possible that neither left nor right pterygoids show the original shape of the quadrate ramus. This area is offset dorsocaudally by an angle of almost 105 degrees with the main axis of the pterygoid, it is flat, if not slightly concave, to overlap the quadrate.

### Ectopterygoid

**Fig 9:**
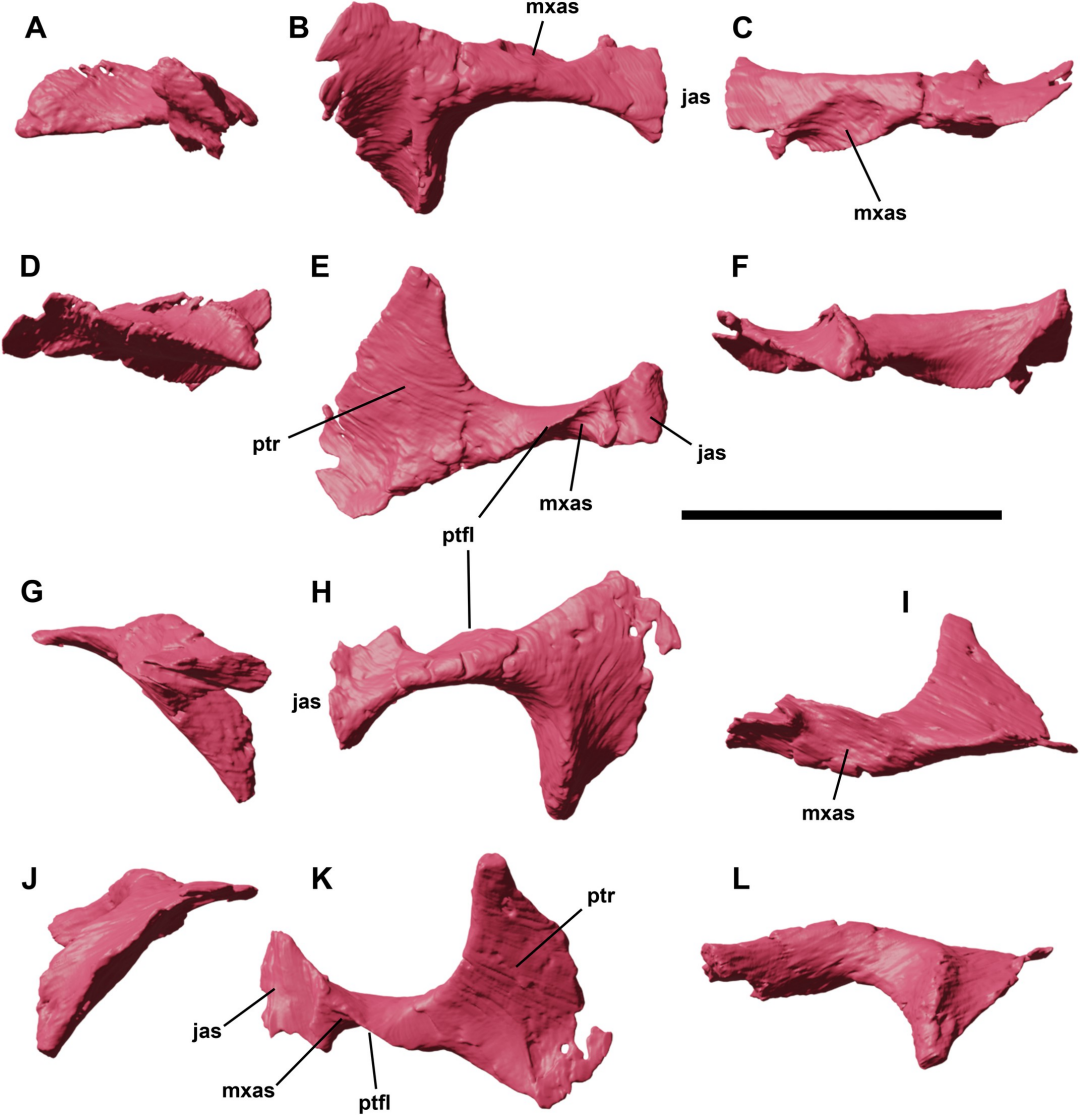
Right and left ectopterygoids of YLSNHM 01942 in lateral (A,G), dorsal (B,H), rostral (C,I), medial (D,J), ventral (E,K) and caudal (F,L) views. Abbreviations: **jas**, jugal articular surface; **mxas**, maxillary articular surface; **ptr**, pterygoid ramus; **ptfl**, pterygoidal flange. Scale bar equals 1 cm.

Both ectopterygoids are preserved in YLSNHM 01942, and still in their natural articulation with the jugals and maxillae (Fig 1K). The ectopterygoid is an axe-shaped element composed of two main parts: a medial, rostrocaudally expanded hatched-like process and a laterally projecting ramus (Fig 9A–9L). The medial process is trapezoidal, caudoventrally curved and slightly concave ventrally to articulate with the transverse flange of the pterygoid. The rostral margin of the medial process is triangular and bulky, whilst its caudal margin has a finger-like caudal end, triangular in cross-section due to a thickened longitudinal ridge along its dorsal surface. The lateral ramus articulated with the medial side of the jugal in a triangular facet. On the rostrodorsal side of the ramus, there is a shallow concavity, bordered by a pterygoideal flange, that limits the mediolaterally expanded and circular articular surface with the caudal end of the maxilla.

### Basisphenoid/parasphenoid

**Fig 10:**
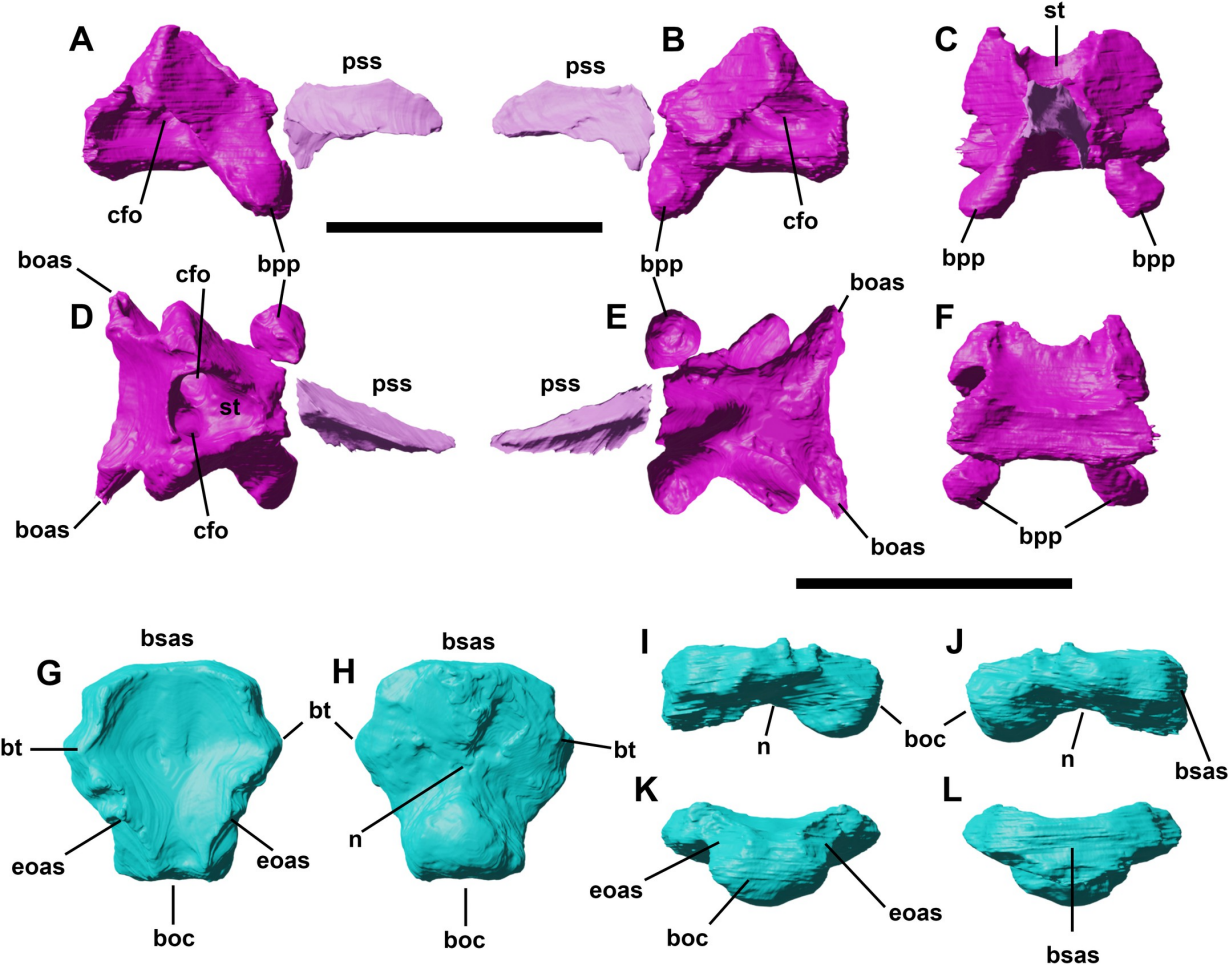
Basisphenoid and parasphenoid of YLSNHM 01942 in right lateral (A), left lateral (B), rostral (C), dorsal (D), ventral (E), and caudal (F) views. Basioccipital of YLSNHM 01942 in dorsal (G), ventral (H), left lateral (I), right lateral (J), caudal (K) and rostral (L) views. Abbreviations: **boas**, basioccipital articular surface; **boc**, basioccipital condyle; **bpp**, basipterygoid process; **bt**, basal tubera; **bsas**, basisphenoid articular surface; **cfo**, carotid aorta foramen; **eoas**, exoccipital articular surface; **n**; notch; **pss**, parasphenoid; **st**, sella turcica;. Scale bars equal 1 cm.

The basisphenoid is almost complete, with only the left basipterygoid process broken and displaced. This element is hourglass-shaped, articulating with the basioccipital along a flat and nearly straight suture (Fig 10A–10F). The basipterygoid processes are short, thick and blunt, almost triangular in cross-section, with a flat side facing laterally. They are separated from each other by a well-developed and deep subcircular recess. Caudal to this separation, the ventral surface of the basisphenoid is flat. Laterally, a large cavity opens on both sides, piercing through the element in two medial circular openings, which correspond to the carotid aortas and are separated by a thin and sharp ridge. These two openings face dorsally within the deep *sella turcica*. In dorsal view, the *sella turcica* has a nearly triangular outline, with thickened margins. Two symmetrical grooves extend between its thickened caudal margin and the prootic articular surfaces. The latter are inclined at about 118° from the longitudinal axis of the basisphenoid, with a flat trapezoidal surface.

A disarticulated element was reconstructed from the rostral area of the skull, near the dentary symphysis, and is tentatively identified as the parasphenoid (Fig 10A). The isolated nature of the element suggests it was not fully fused to the basisphenoid due to the young stage development of YLSNHM 01942. The articular surface between the two bones cannot be precisely traced back because of erosion of their margins. The parasphenoid is an elongated, stout element that is horizontally projected from the main axis of the basisphenoid. It has a V-shaped transverse section, with a strong ventral keel and two lateral wings that are separated by a central canal deepening caudally, in continuity with the hypophyseal cavity.

### Basioccipital

The basioccipital is a small, wide and flattened element still in articulation with other neurocranial bones (Fig 10G–10L). The occipital condyle is enlarged and dorsoventrally thickened, projecting mostly vertically, with a smooth and slightly flattened surface. Dorsally, a wide U-like depression forms the ventral side of the foramen magnum. The ventral flange reported in the holotype and present in other neornithischians (*Hypsilophodon*, *Orodromeus*, *Yinlong*, *Zephyrosaurus*, *Changchunsaurus* and *Haya* [3, 4, 16]) is faintly visible in YLSNHM 01942, and is perhaps an ontogenetic feature. Rostral to the condyle, the basioccipital expands transversely to form the basal tubera, separated from the occipital condyle by a deep basioccipital neck. The basal tubera are not as dorsoventrally enlarged as the occipital condyle, instead they maintain a constant thickness until they reach the midline of the basioccipital. The basal tubera are separated by a shallow notch, as reported by Barrett and Han [3]. The endocranial floor is concave and a faint median ridge extends rostrocaudally from the area of maximum width of the basioccipital (corresponding to the expansion of the basal tubera) up to its rostralmost margin in contact with the basisphenoid. This ridge might correspond to the “crest” reported in the sauropodomorph *Macrocollum itaquii* [32], although it does not share the same keen morphology. The articular facets for the exoccipitals are located dorsolaterally, above the occipital condyle, and they have an elliptical shape.

### Exoccipital/opisthotic

**Fig 11:**
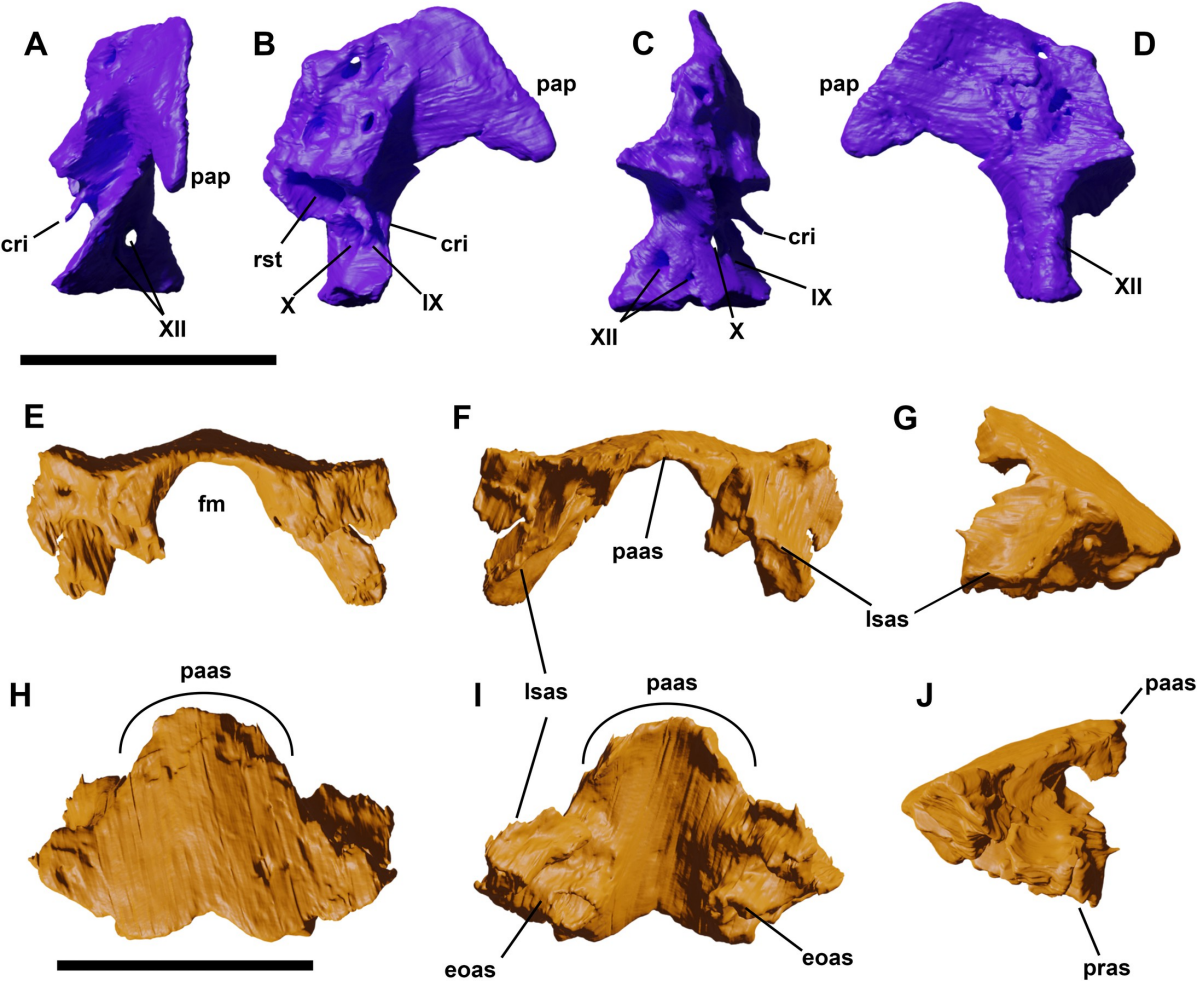
Left Exoccipital-ophistotic of YLSNHM 01942 in lateral (A), rostral (B), medial (C) and caudal (D) views. Supraoccipital of YLSNHM 01942 in caudal (E), cranial (F), left lateral (G), dorsal (H), ventral (I), and right lateral (J) views. Abbreviations: **cri**, crista interfenestralis; **eoas**, exoccipital articular surface; **fm**, foramen magnum; **lsas**, laterosphenoid articular surface; **pap**, paroccipital process; **paas**, parietal articular surface; **pras**, prootic articular surface; **rst**, recessus scalae tympani. The Roman lettering corresponds to the foramina for the cranial nerves. Scale bar equals 1 cm.

**Fig 12:**
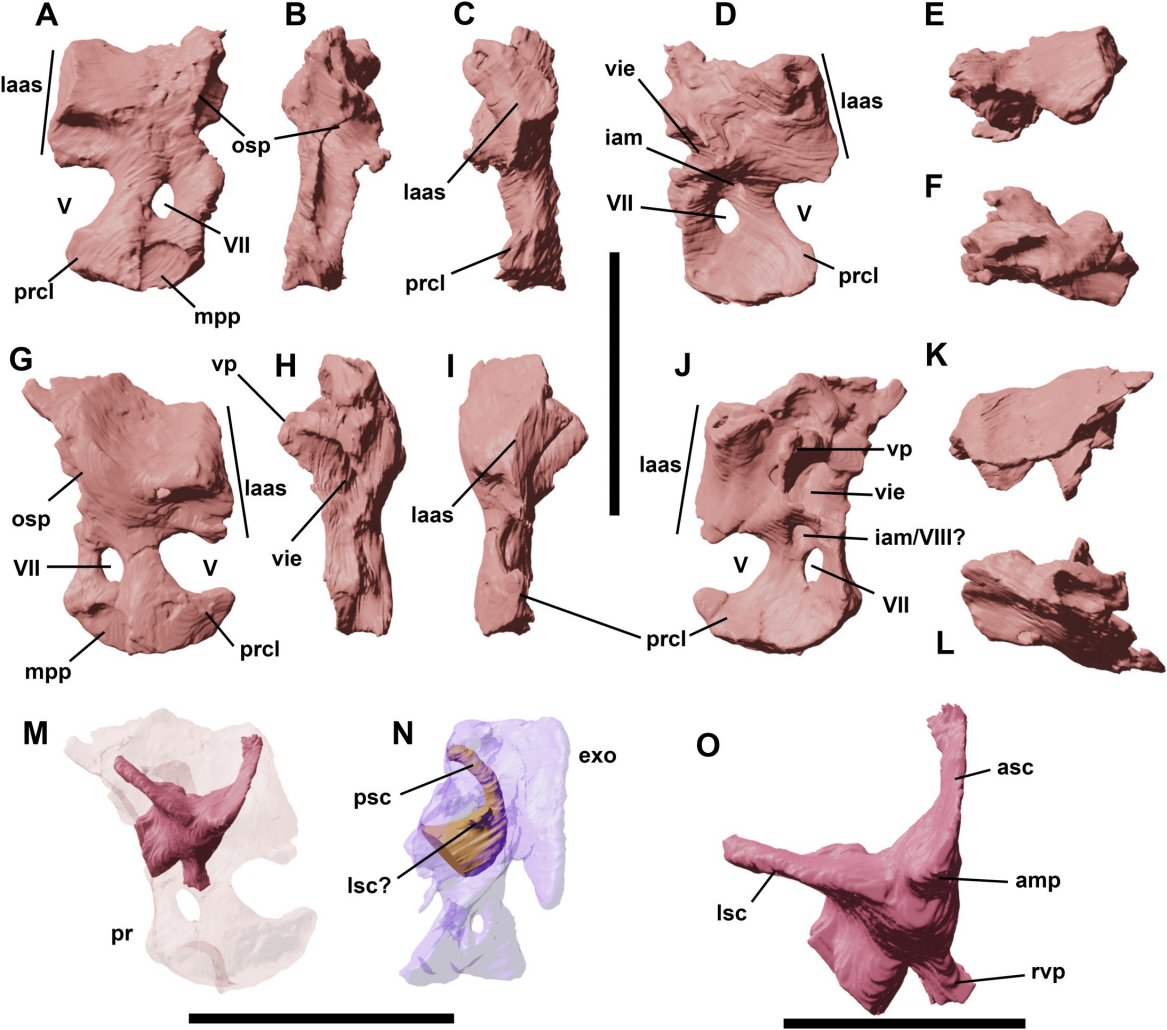
Left (A-F) and right (G-L) prootics of YLSNHM 01942 in lateral (A,G), caudal (B,H), rostral (C,I), medial (D,J), dorsal (E,K), and ventral (F,L) views. Reconstruction of the endosseous labyrinth within the right prootic (M) with a close-up of the model (O), and left exoccipital/ophistotic (N) in lateral views. Abbreviations: **amp**, ampulla; **asc**, anterior semicircular canal; **exo**, exoccipital/ophistotic; **iam**, internal acoustic meatus; **laas**, laterosphenoid articular surface; **mpp**, depression for *M*. *protactor pterygoideus;* **osp**, otosphenoidal crest; **pr**, prootic; **prcl**, *processus clinoideus*; **psc**, posterior semicircular canal; **vie**, vestibule of inner ear; **vp**, vestibular pyramid. Scale bars equals 1 cm and 0.5 cm (A-N) (O).

As in other neornithischians, the suture between the exoccipital and ophistotic is not visible [29]. In YLSNHM 01942, only the left one is preserved, slightly displaced from its original location (Figs 1K and 11A–11D). Half of the articular surface with the supraoccipital is clearly visible in proximal view, whilst the other half is in contact with the bone. Caudoventrally, the exoccipital thickens into the condyloid, which forms the lateral side of the foramen magnum, and it articulates with the basioccipital. The main axis of the exoccipital/opisthotic is slightly twisted mediolaterally, with a broad and well developed paroccipital process that directs laterally. This broad, trapezoidal shelf is not pendant, but it expands craniocaudally to form a ventrally directed subtriangular process in the distal region. On the rostrolateral surface, a rod-like process (*crista interfenestralis*) projects rostrally, like in *Hypsilophodon* [18], and borders dorsally two foramina. Rostral to this elongated process, the *recessus scalae tympani* is a wide and circular opening piercing the dorsal region of the exoccipital. The channel is further divided into a dorsomedial and a dorsolateral chamber, separated by a thin ridge. Segmentation reveals a large initial chamber that separates at the ridge, forming a dorsolateral short boss-like chamber whilst the longer and tubular dorsomedial passage curves rostrally (Fig 12M–12O). Four foramina pierce the condyloid from side to side. The caudal ones represent the passages for the two branches of c.n. XII, while the other two, the opening for c.n. X and the rostral one for c.n. IX, are situated next to the *crista interfenestralis* [4, 18, 33]. The last one is situated rostral to the one for the IX cranial nerve, and it might have served as a separate passage for the IX nerve [33], or the internal jugular vein [34]. In the referred specimens, Barrett and Han [3] reported only one opening for c.n. XII in *Jeholosaurus*, suggesting a certain intraspecific variation for the developments of these foramina. On the medial surface of the base of the paroccipital process, a series of cavities open, some of them piercing through the entire body of the exoccipital. The nature of these openings might be vascular, as there are no reports of other cranial nerves piercing in this region.

### Supraoccipital

The supraoccipital in YLSNHM 01942 is well preserved (Fig 11E–11J), and it offers new insight on its anatomy, since the supraoccipitals are not present or poorly preserved in the holotype and referred materials [3]. It is a trapezoidal element, which contacts the parietal dorsolaterally, whilst the exoccipital/opisthotics articular surfaces cannot be ascertained as these elements are dislocated. It is inclined caudally at an angle of 120 degrees with the main axis of the skull. The dorsal contact between supraoccipital and the caudomedial sides of the paired frontals (the midpoint of the sagittal crest) is not fused, leaving a small, narrow opening. The lateral wings of the supraoccipital extend laterally. The ventral border is smoothly concave, forming the dorsal margin of the foramen magnum, as in basal ornithopods [29]. The external surface of the supraoccipital is characterized by a well-developed, dorsoventrally expanded bump (or ridge as by [3, 4, 18]) along its midline, originating from the dorsal contact with the frontal, but not reaching the ventralmost margin of the supraoccipital. In caudal view, the wings thicken, forming the articular surface for the exoccipital/opisthotics. In ventral view, the dorsal surface of the basioccipital is smooth and slightly arched. The articular surfaces for the prootics, laterosphenoids and exoccipital/opisthotics are rough and pierced by vascular foramina.

### Prootic

Both prootics are identified via segmentation, and their morphology ‐ missing in Barrett and Han [3] ‐ can be described in detail. The right element seems to be better preserved than the left (Fig 12A–12L), therefore the anatomical description is based on the former. The prootic is an irregularly shaped bone that forms the lateral wall of the braincase, articulating with the laterosphenoid cranially, the basioccipital ventrally, the supraoccipital dorsally, and the exoccipital/ophistotic caudally. A large and roundish foramen opens on the lower half of the element, corresponding to the *foramen ovale* for cranial nerve (c.n.) VII. This foramen is relatively larger than in *Hypsilophodon foxii* and *Lesothosaurus diagnosticus*. The ventral region of the prootic has a curved outline, tapering to a rostrally-directed *processus clinoideus* [*sensu* 35], which forms the ventral margin of the large rostral opening for cnV, together with the missing laterosphenoid. In the laterocaudal region, there is a depression likely corresponding to the attachment site for *M*. *protactor pterygoideus*. A long otosphenoidal crest extends along the caudal margin of the prootic, as in *Lesothosaurus*, from the posterodorsal region down to the basipterygoid process [24, 36]. In medial view, there is a foramen dorsal to the foramen for c.n. VII that might correspond to the c.n. VIII or the internal acoustic meatus [*sensu* 24]. It opens into a large, medial chamber, the vestibule of the inner ear, bordered dorsally by a medially expanded vestibular pyramid. From the opening of the vestibule, the inner cavity curves rostromedially, exiting from a small foramen on the mediorostral side of the prootic, close to the articular surface with the laterosphenoid (Fig 12M–12O). It corresponds to one of the branches of the semicircular canal of the inner ear, that is also preserved inside the left prootic.

### Predentary

Only the ventral process of the predentary is preserved (Fig 1F). It is flattened caudally and cup-shaped cranially to form the mandibular symphysis. The caudal end of the ventral process is unilobate and slightly expanded mediolaterally, as reported in other *Jeholosaurus* specimens [3], even though its caudal margin forms a faint middle invagination, as in *Changchunsaurus* [16]. The preserved rostral portion is developed dorsally and tapers rostrally. The right lateral side of the rostral portion has a faint concavity. The dorsal half of the element is stouter and expanded medially.

### Dentary

**Fig 13:**
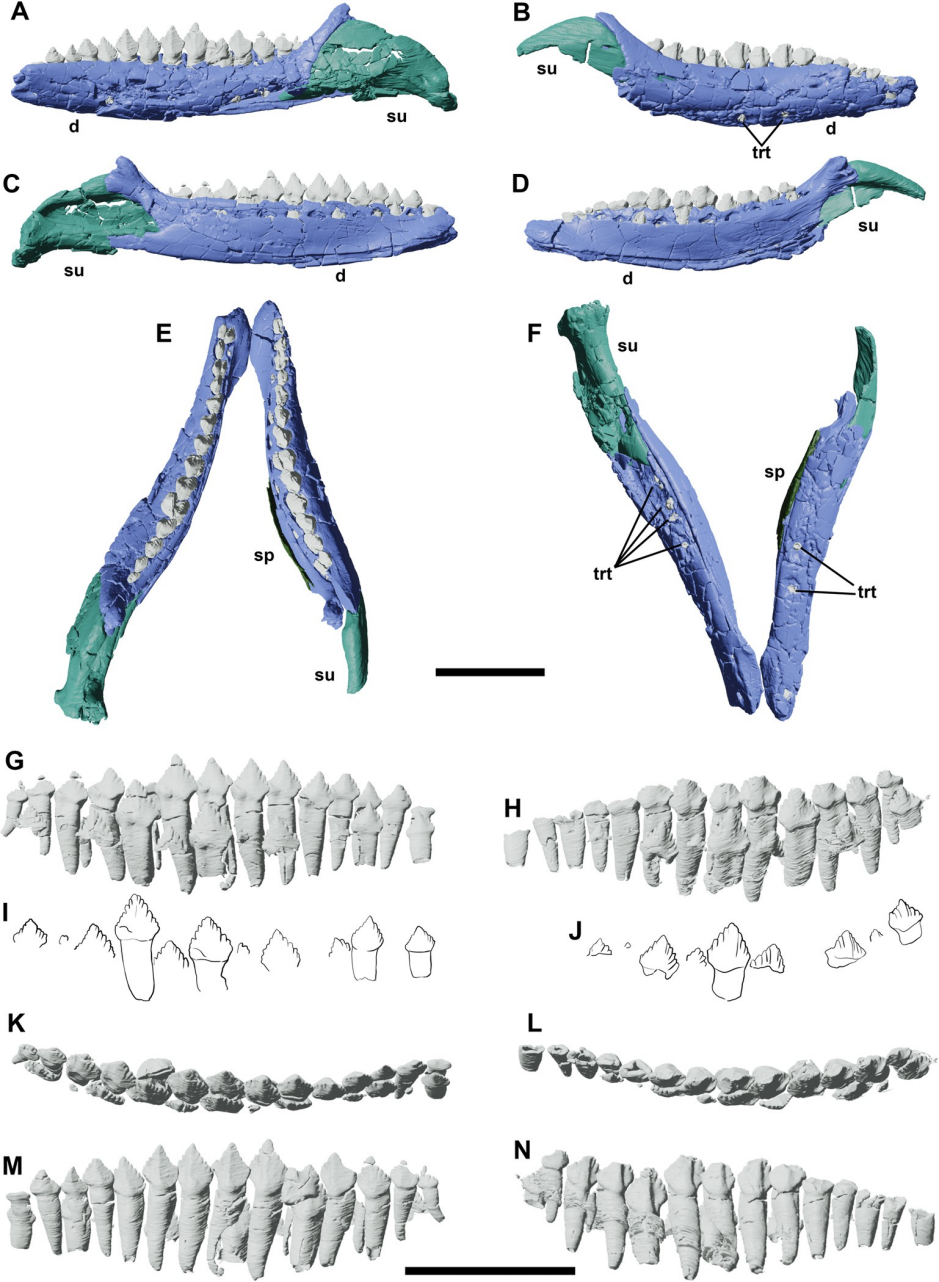
Left and right lower jaws of YLSNHM 01942 in lateral (A,B), medial (C,D), dorsal (E) and ventral (F) views. Left and right dentary teeth in lingual (G,H), dorsal (K,L), and labial (M,N) views. The drawn teeth represent the replacement teeth in the dentaries in lingual view (I,J). Abbreviations: **d**, dentary; **sp**, splenial; **su**, surangular; **trt**, teeth roots. Scale bars correspond to 1 cm.

**Fig 14:**
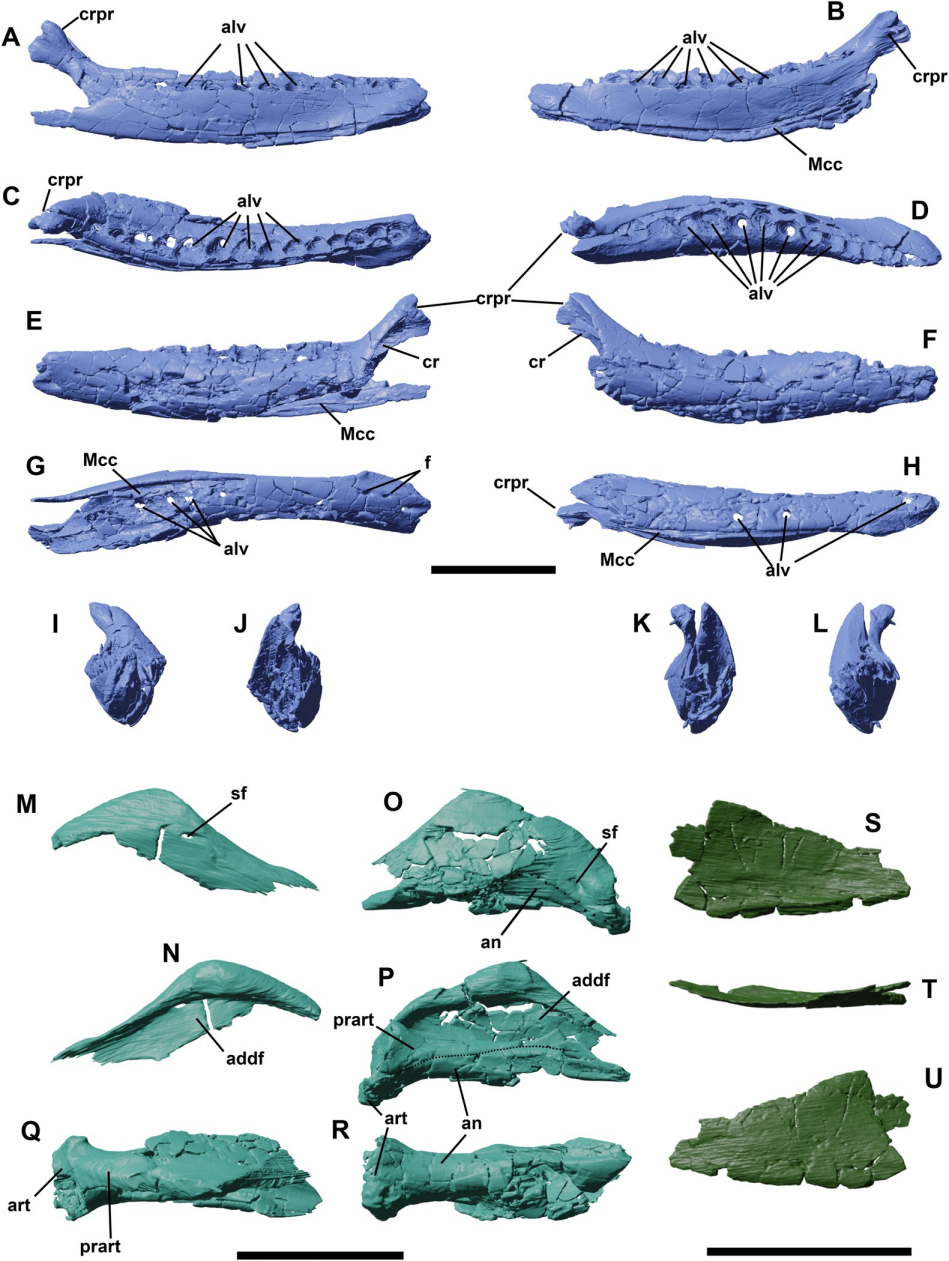
Left and right dentaries of YLSNHM 01942 in lateral (A,F), lingual (B,E), dorsal (C,D), ventral (G,H), rostral (I,K), and caudal (J,L) views. Right surangular in lateral (M) and medial view (N). Left surangular/angular complex in lateral (O), medial (P), dorsal (Q), and ventral (R) views. Right splenial in lateral (S), dorsal (T), and lingual (U) views. Abbreviations: **addf**, adductor fossa; **alv**, alveoli; **an**, angular; **art**, articular; **cr**, coronoid; **crpr**, coronoid process; **Mcc**, Meckelian canal; **prart**, prearticular; **sf**, surangular foramen. Scale bars equal 1 cm.

Both dentaries are present in YLSNHM 01942 at different degrees of preservation (Figs 13A–13F and 14A-14H). The teeth are also present, although those in the left dentary are overlapped by the maxilla and the maxillary teeth. In lateral view, a total of 14 teeth can be seen (Fig 13G and 13M), although the first four in the right dentary are truncated. As similar as the maxillary teeth, the dentary tooth roots pierce through the height of the bone, with their distal tips extending out from the ventral side of the dentaries (Fig 13F). The dentary is a long and slender bone, but, in contrast with the *Jeholosaurus* dentaries described by Barrett and Han [3], their ventral side is not straight but rather convex in lateral view. The dorsal and ventral margins of the dentary are parallel to each other along the main shaft, then taper rostrally to contact the predentary. A very short diastema is present in this area. In ventral view, the rostromedial side of the dentaries is slightly curved medially to form the dentary symphysis, whilst a flat and rugose surface develops ventrally for the caudoventral process of the predentary. The lateral side of the dentary is dorsoventrally convex; the tooth row is inset and separated from the lateral outline of the dentary by a buccal platform. The latter is contiguous with the main shaft of the dentary, lacking the prominent ridge described in *Changchunsaurus* [16], or even the reduced ridge in other *Jeholosaurus* specimens [3] and *Changmiania* [13]. A series of numerous foramina open on the lateral side of the dentary rami. The caudal portion of the dentary curves dorsally to partly form the stout and blunt coronoid process, forming an oblique contact with the surangular. The dentary contribution to the coronoid process consists in a small subtriangular process that lies on the lateral side of the coronoid process of the surangular. In YLSNHM 01942, this contact is not fully fused. The medial side of the dentary can be described in detail thanks to the preservation of the specimen. A long and shallow Meckelian canal extends close to the ventralmost border of the medial side of the dentary, covered caudally by the splenial. As in *Changchunsaurus* [16, 37], a shallow groove extends parallel to the main axis of the dentary, below the tooth row in lingual view, bearing well-developed slit-like replacement foramina.

### Surangular

The left surangular is particularly well preserved in YLSNHM 01942 (Fig 14O–14R); the right one is much more fragmentary (Fig 14M and 14N). The surangular forms the caudodorsal margin of the well-developed coronoid eminence and approximately half of the height of the mandible caudal to the dentary, although the rostral part of the surangular/angular suture is difficult to trace. A small foramen appears to be present on its lateral surface of the surangular adjacent to the apex of the coronoid process of the dentary, as is often present in other basal ornithischians (e.g., *Hypsilophodon* [18], *Changchunsaurus* [16]). Its lateral is slightly convex dorsoventrally and devoid of the rostrocaudally extending ridge observed in basal ceratopsians on the lateral surface of the surangular, in contrast to the condition in some basal ceratopsians (e.g. *Archaeoceratops*: IVPP V11114). Medially, the surangular forms the lateral wall and dorsal roof of the adductor fossa.

### Angular

In lateral view, the angular forms the ventral half of the caudal part of the mandible (Fig 14O and 14P). It articulates dorsally with the surangular and its lateral surface is gently convex dorsoventrally. Its ventral margin is strongly concave in lateral view. Caudally, it participates in the ventral surface of the retroarticular process. Medially it articulates dorsally with the prearticular and is medially overlapped at its rostral end by the splenial. Only a small portion of the angular is therefore visible in medial view.

### Coronoid

The dorsal process of the coronoid is lobate and set medial to the coronoid process of the dentary (Fig 14E and 14F), contacting the rostrodorsal border of the surangular, as also observed in *Hypsilophodon* [18] and *Changchunsaurus* [16]. It extends rostrally immediately ventral to the last erupted teeth on the medial side of the mandible.

### Splenial

The right splenial covers the medial side of the dentary and has a flat subtriangular shape (Fig 14S–14V). Its ventral side extends parallel to the ventral margin of the dentary and overlaps the caudal portion of the Meckelian canal. Its rostral end tapers, even though the triangular tip is broken off. The caudal portion of the splenial is differentiated into a dorsal process that extends to the level of the dentary tooth row, and a caudal process that ends at the level of the base of the coronoid process. Another dislocated fragment was recognized as a partial left splenial, close to the dentary symphysis.

### Prearticular

The left prearticular is preserved in articulation on the medial side of the mandible (Fig 14P and 14Q). It is a dorsoventrally narrow, transversely compressed bone that participates in the ventromedial margin of the adductor fossa. Caudally, it expands dorsoventrally and forms part of the cup for the articular. Its rostral end also expands dorsoventrally to articulate with the caudal end of the dentary and is overlapped by the splenial. Like in *Changchunsaurus* [16], there is no trace of an internal mandibular foramen.

### Articular

The left articular is preserved, but its precise limits with the surrounding bones are difficult to discern (Fig 14P–14R). This bone sits in a cup formed by the prearticular medially, the angular ventrally, and the surangular laterally. It presumably formed most of the jaw articular surface.

### Maxillary teeth

A total of 11, perhaps 12, maxillary teeth are present in YLSNHM 01942 (Fig 2J and 2K), whilst there are 12–15 teeth in IVPP V12529 [3], up to 18 in older specimens [38], 15 in *Changmiania* [13], 15–16 in *Haya* [4, 9], and 10–11 in *Hypsilophodon* [18]. However, an empty alveolar socket on the left maxilla suggests a total of 13 teeth in this specimen as in the late juvenile IVPP V12530 [38]. The teeth are large, peg-like, with a slight caudal orientation. The rostrally located teeth are smaller, and they reach the maximum apicobasal and mesiolateral lengths in the middle point of the maxilla, corresponding to teeth 7–9. They reduce their size again distally. They are imbricated, meaning that the distal end of each crown laterally overlaps the mesial part of the succeeding one (as in *Haya* [4]), and there is one singular functional tooth per alveolus. A thick and enlarged cingulum separates the crown from the root. Although the proportions of the crown vary within the dental battery, the height/weight ratio of the crown is about 1. As reported in the holotype, the maxillary teeth lack a primary ridge and present a central eminence that extends from the marginal tip to the center of the lingual surface. The crown surface bears several denticles, triangular in shape, with a maximum count of 9 or 11. However, in most teeth, denticles cannot be counted because of tooth wear that smoothed the crown margins. The denticles correspond to a series of secondary ridges that furrow the lingual side of the crown, and, like the central eminence, they extend towards the cingulum, stopping in the middle of the lingual surface. A series of developing teeth is visible in the segmented reconstruction, seven on the left, and three on the right, at different developmental stages, and there seems to be one single replacement tooth per alveolar position.

### Dentary teeth

There are 13–14 dentary teeth, resembling the maxillary teeth, although their crown looks more lanceolate (Fig 13G–13N). This count is within the juvenile range described by Hu et al. [38], with up to 17 dentary teeth in older individuals. As per the maxilla, there is a single active tooth per alveolus, with a possible single replacement tooth, different from *Jeholosaurus* IVPP V15717 that shows a second generation of replacement teeth [38]. On the left dentition, one tooth has almost completely replaced the old one, for which only the distal part of the crown remains. The cingulum is stout and the roots are slightly curved rostrally. The most evident difference with the maxillary teeth is the stronger expansion of the central eminence on the labial side of the crown, almost resembling the primary ridge present in more derived iguanodontians. Each secondary ridge ends in a denticle on the crown margin, and a total of 12 denticles are counted on the best-preserved teeth. Nine replacement teeth are present on the left dentary, whilst the right one bears 12 at different growth stages.

### Phylogenetic analysis

**Fig 15:**
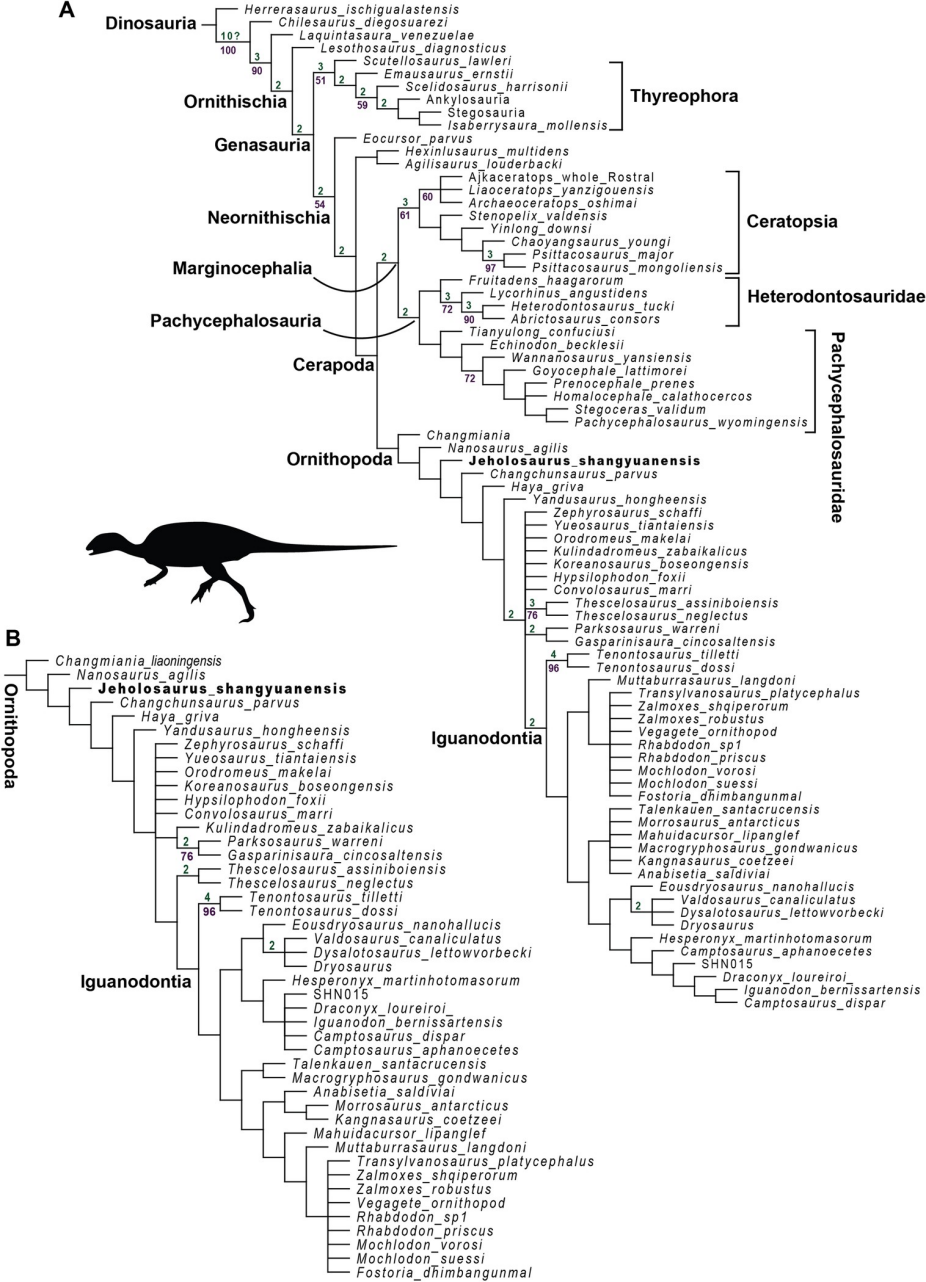
Strict consensus tree of the parsimony analysis, equal weighting (A) and extended implied weighting (B), based on Dieudonné et al (2021), with the inclusion of *Changmiania*, *Ajkaceratops* and the new scoring of *Jeholosaurus*. Numbers over branches indicate bootstrap values (in green), while numbers below branches indicate Bremer supports (in purple). Silhouette of *Jeholosaurus* from Phylopic.

In our analysis, we solved previously unscored characters for *Jeholosaurus* as following: lacking a prominent anterolateral boss in the maxilla articulating with the medial premaxilla (31:0); dorsally flattened frontals over the orbit (46:1); jugal wing with a greater height than the quadrate (78:0); straight posterior maxillary process (82:0); presence of a deep groove on the ectopterygoid articular facet of the jugal (83:1); absence of the fossa on the pterygoid ramus of the quadrate (102:0); absence of the Y-shaped indentation in the ophistotic (106:0); trigeminal nerve bounded by prootic and laterosphenoid (107:0); absence of the prootic-basisphenoid plate (108:0); dorsal margin of the foramen magnum formed by the supraoccipital with a small participation of the basioccipital (109:0); endocranial cavity roofed by the supraoccipital, overlaying the ophistotic (111:1); *foramen magnum* occupies less than 50% of the occipital condyle (114:0); absence of an anteroposteriorly directed groove on the ventral surface of the basioccipital (116:0); equal depth for the basal tubera of the basioccipital (119:0), at the level of the basioccipital condyle (120:0); contact between pterygoid and maxilla posterior to the tooth row (127:1); bowed tooth row in the dentary, aligned to the coronoid process and located anteriorly to it (146:0); absence of wear facets on teeth (167:0), showing symmetrical enamel (170:0); maxillary teeth bearing small secondary ridges on maxillary teeth (171:1), lingually concave crown (176:0), a central apical ridge (179:0) and straight roots (182:0); <10 dentary teeth (187:0) with central apical ridge (186:0), evenly-spaced and parallel secondary ridges (190:0), present on both labial and lingual side of the crowns (191:0), and straight roots (192:0). Character 149 describes the number of dentary teeth, with a specific number limit between 11–13 (character code 1) and 14–17 (character code 2). In YLSNHM 01942 the left dentary bears 14 teeth, whilst only 13 are recognized in the right one. Given the absence of the rostral portion of the dentaries, we hypothesize the first alveolus in the right element is missing, scoring for the final count of 14 dentary teeth. The inclusion of the amended scoring for the previously unknown characters of *Jeholosaurus* changed the position of the taxa. We run the analysis with the original scoring using the same methodology described in this paper, and *Jeholosaurus* was found as sister taxon of *Changchunsaurus* within a clade with *Haya griva*. The inclusion of the new characters collapsed this node and created the polytomy of *Zephyrosaurus*, *Yueosaurus*, *Orodromeus*, *Kulindadromeus*, *Koreanosaurus*, *Hypsilophodon*, *Convolosaurus*, *Theschelosaurus*, *Parksosaurus* and *Gasparinisaura*. The basalmost ornithopod found by the original analysis is *Changmiania*. With the new scored characters, the equal weighting (EW) analysis returned a topology of the strict consensus tree (836 trees found, with a tree length of 1264), finding *Jeholosaurus* near the base of Ornithopoda (Fig 15A). *Changmiania* is still placed as the basalmost ornithopodan in a well-resolved topology, followed by *Nanosaurus*. In contrast to Yang et al. [13], *Changchunsaurus* does not result as the sister taxon of *Jeholosaurus*, as found in our test of the original scoring as well. The extended implied weighting analysis (k = 15) shows a similar result at the base of Ornithopoda (10 trees found, with a tree length of 1274), with the same affinities for *Changmiania*, *Nanosaurus*, *Jeholosaurus*, *Changchunsaurus*, *Haya* and *Yandusaurus* (Fig 15B). Both equal weighting and extended implied weighting analyses present a Consistency Index of 0.286, a Retention Index of 0.610 and a Rescaled Consistency Index of 0.174. The positioning of *Jeholosaurus* within Ornithopoda is supported the following characters: rugose surface over the anterior and dorsal surfaces of the premaxillae (13: 0>1), a fossa-like depression in the premaxilla-maxilla contact (27: 0>1), the exclusion of the jugal from the posteroventral margin of the external antorbital fenestra by lacrimal–maxilla contact (37: 0>1), narrow and elongated frontals (45: 0>1), grooves on either side of midline on the anterior surface of the predentary (133: 0>1), and a broadly swollen ischiadic peduncle of the ilium that projects ventrally (274: 0>1). The positioning of *Jeholosaurus* with *Changchunsaurus* and the more derived ornithopods is supported by an overlapping of adjacent crowns with an overlapping “en échelon” pattern of the maxillary and dentary teeth (174: 0>2) and a moderate anterior extension of the greater trochanter beyond the femoral head (297: 0>2).

## Discussion

**Fig 16:**
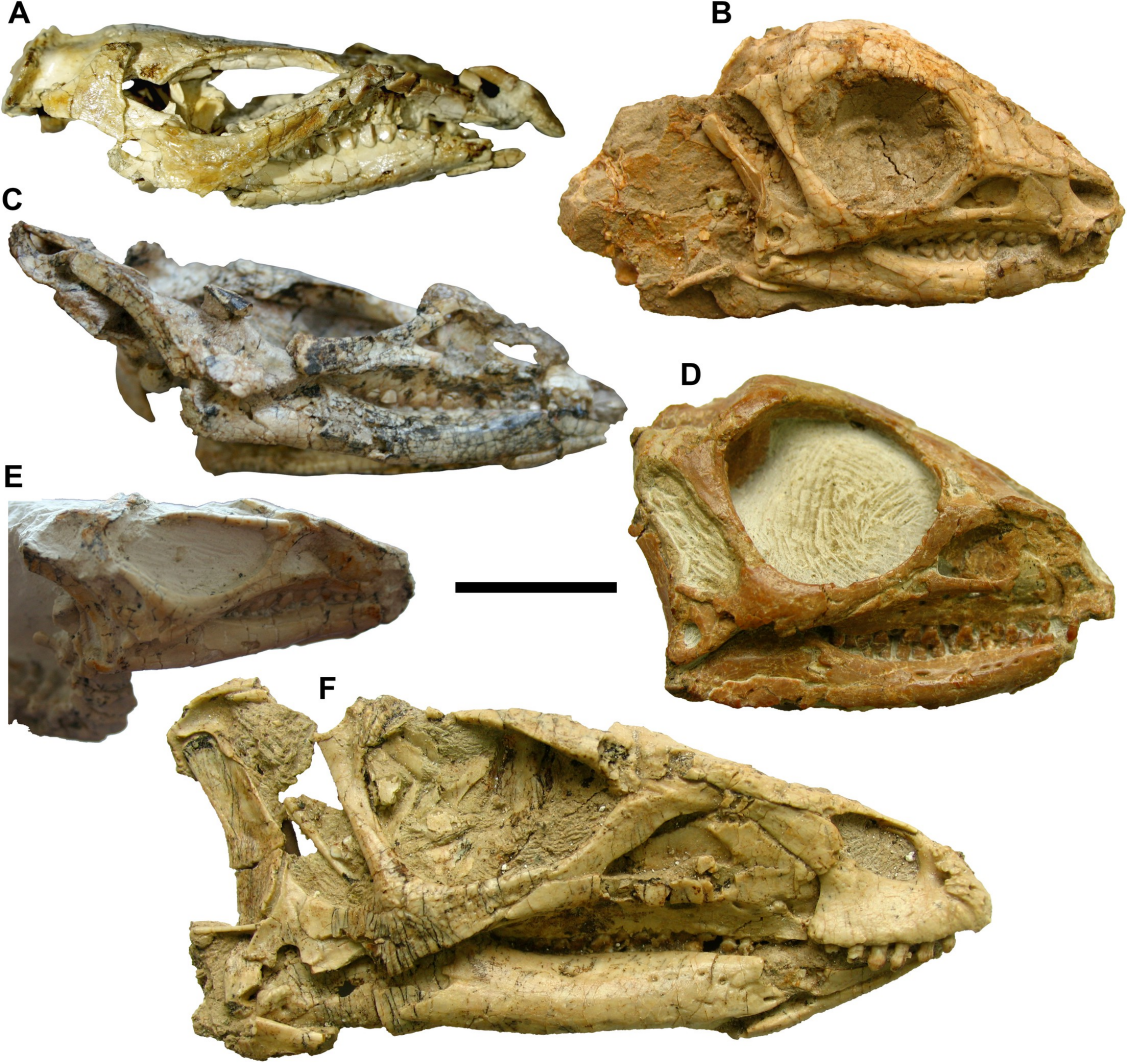
Published *Jeholosaurus* skulls: YLSNHM 01942 (A), IVPP V12530 (B), IVPP V12529(C), IVPP V15718 (D), IVPP V15719 (F), and IVPP V15716 (F). The pictures of specimens B-F are kindly provided by Dr. Paul Barrett (NHMUK). Scale bar = 2 cm.

**Fig 17:**
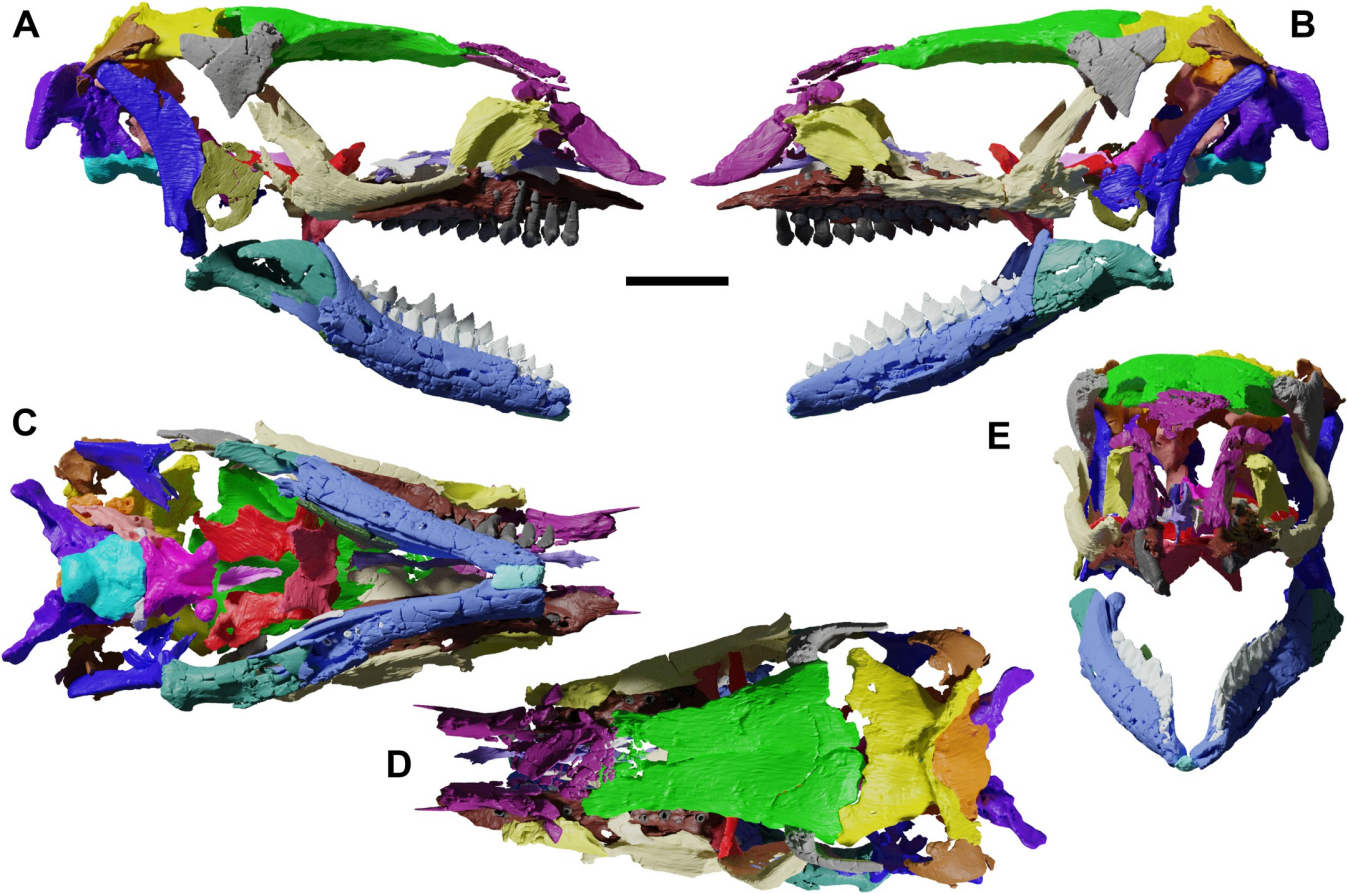
Hypothetical assemblage of YLSNHM 01942 in left lateral (A), right lateral (B), ventral (C), dorsal (D) and rostral (E) views, following other skulls in Fig 16, and cranial reconstruction for *Hypsilophodon* [18]. Scale bar = 1 cm.

The dorsoventrally flattened and crushed appearance of the skull in YLSNHM 01942 resembles the condition in IVPP V12529, V15716, V15717 and V15719, contrasting with the dorsoventrally expanded and roundish outline in IVPP V12530 and V12518 (Fig 16). The compression of the specimens might be related to the taphonomical environment of *Jeholosaurus*. The specimens are recovered from the tuffaceous sandstone of the first member of the lower Yixian Formation [1], but further information on the geological status of the fossiliferous layer is missing from the literature. We re-assembled the skull following the more complete specimens (Fig 17), confirming that the orbital diameter is about 40% the length of the skull (perhaps lower given the absence of the rostral portion of the premaxillae).

The early ontogenetic stage of YLSNHM 01942 is suggested by i) its relatively small size; ii) the unfused cranial elements; iii) its wide orbital diameter; iv) a strongly vaulted skull roofs; and v) the absence of a nodular ornamentation on the jugal and postorbital [3]. Barrett and Han [3] suggested another ontogenetic feature, that is the contact between the premaxilla and lacrimal, but because of the poor preservation of the articulation in YLSNHM 01942, we cannot confirm the validity of this character in this specimen. The unique diagnostic character that Barrett and Han [3]included in the diagnosis is the presence of the quadratojugal foramen. This feature is also present in *Haya griva* [4, 9], but in *Jeholosaurus* YLSNHM 01942 is larger, while in *Haya* is reduced to a small opening [4, 9]. A row of foramina might be autapomorphic for *Jeholosaurus* [3], and the presence of one foramen in the supposed nasal of YLSNHM 01942 might be indicative of that, considering the lower number of foramina as an ontogenetic feature. The early juvenile stage and intraspecific variation might explain the series of anatomical variations we detected in this specimen compared to other *Jeholosaurus* skull described by Barrett and Han [3]: i) the flat (*vs* concave) lateral surface of the quadratojugal (lateral) wing (Fig 6C), ii) the sub-squared quadratojugal, not elongated and slender, missing of the ventrolateral tab-like process, showing the quadratojugal foramen in a more caudal position (Fig 6L); iii) the parietals bearing a small faint bump each, located rostrally to the caudolateral processes (Fig 5G); iv) the transverse flange of the pterygoid is not thicker dorsoventrally towards its distal end (Fig 8); v) higher count of cranial openings in the exoccipital/ophistotic (Fig 11A–11D); vi) convex ventral side of the dentary (Fig 14A and 14B); vii) buccal platform contiguous with the main shaft of the dentary, lacking a reduced ridge (Fig 14C and 14D). Furthermore, YLSNHM 01942 shows 13 maxillary teeth with only one generation of replacement teeth and 13/14 dentary teeth as in other juvenile *Jeholosaurus* described by Hu et al. [38] (2024). *Jeholosaurus* lived together with another ornithopod, *Changmiania*, but this latter taxon differs for lacking a sagittal crest in the parietal, the presence of prominent caudal boss at the dorsolateral corner of the squamosal, and a squamosal with a straight and rostrocaudally elongated rostral process, features absent in YLSNHM 01942.

### Phylogenetical position

The inclusion of *Changmiania* and the new scoring for *Jeholosaurus* resulted in a stable base for Ornithopoda, both in the equal weighting and extended implied weighting analyses (Fig 14). Both Dieudonné et al. [8] and Rotatori et al. [11] performed the analysis without considering *Changmiania*, and such exclusion produced a large polytomy at the base of Ornithopoda. Recently, Czepiński and Madzia [14] re-evaluated the enigmatic materials of *Ajkaceratops*, an ornithischian dinosaur from the Late Cretaceous of the western Tethyan archipelago [39]. The authors provided a scoring for the species considering it either a basal ceratopsian or a basal neornithischian. In our analysis, we followed the first hypothesis, considering the “beak” of *Ajkaceratops* as formed by the rostral bone. Interestingly, if the specimen of *Ajkaceratops* is considered as a premaxilla, *Ajkaceratops* is detected within Ornithopoda as a sister taxon of *Changmiania liaoningensis* (S2 Fig). The extended implied weighting analysis results in a topology similar to the equal weighting, with a few considerations. In the K = 15, the node of *Thescelosarus assiniboiensis* and *Thescelosaurus neglectus* is the sister taxon of Iguanodontia, and *Kulindadromeus* is in a close relationship with the node formed by *Parksosaurus warreni* and *Gasparinisayra cincosaltensis* (Fig 15B). The relationships within Elasmaria are better solved, but the clade is found to be polyphyletic, with the ‘elasmarian’ taxa on an evolutionary pathway towards Rhabdodontomorpha. This result is compelling, but we consider it with attention given the paucity of specimens, and the actual disagreement over the positioning of Elasmaria in different phylogenies [6, 40].

### Cranial nerves and endosseous labyrinth in *Jeholosaurus*

Whilst paleoneurological descriptions have been proposed for hadrosaurids [41], information on cranial nerves, endosseous labyryntolfactory tracts and cranial endocasts in basal neornithischians are still poorly understood. Endocasts have been described in *Thescelosaurus neglectus* [42], *Dysalotosaurus lettowvorbecki* [43] and *Hypsilophodon foxii* [18], whereas possible comparative materials from early ceratopsians are described in *Psittacosaurus luijatunensis* [44], *Auroraceratops* sp. [45] and *Liaoceratops yanzigouensis* [46].

It was not possible to reconstruct the original shape of the endocast in YLSNHM 01942 because of the disarticulation and deformation of the cranial elements, as well as lack of important elements such as the laterosphenoids. However, the cranial nerve openings and parts of the endosseous labyrinth in the exoccipitals/ophistotic and prootics are nicely preserved in YLSNHM 01942.

Only two openings are recognized in the prootics, here hypothesized belonging to c.n. V and c.n. VII. Both are enlarged, the space for c.n. V larger than the other. c.n. VII is the facial cranial nerve, located entirely within the prootic (Fig 12), between the trigeminal nerve (c.n. V) and the *fenestra vestibuli* as in sauropsids [47]. The facialis foramen is relatively larger than in *Thescelosaurus neglectus* and *Dryosaurus*. c.n. V is located on the rostral margin of the prootic and it opens towards the contact with the missing laterosphenoid, suggesting a strong participation of this bone in the formation of the margin of c.n. V as in *Thescelosaurus assiniboiensis* [26] and *Euparkeria* [48]. In contrast, the trigeminal nerve (c.n. V) is completely bordered by only the prootic in *Dryosaurus* [49], *Dysalotosaurus* [43] and *Thescelosaurus neglectus* [26].

Four foramina are in the proximal portion of the exoccipital-ophistotic (Fig 11), but the assignment to cranial nerves is complicated by the young ontogenetic stage of YLSNHM 01942. Only three foramina are recognized in *Thescelosaurus neglectus* [26] and *Dysalotosaurus* [43], two-three foramina in *Haya* [9] and only two in *Dryosaurus* [49]. Moving caudorostrally, we identified the foramina for c.n. XII, c.n. X and c.n. IX in YLSNHM 01942. The hypoglossal nerve branches into two, exiting from the two caudally located foramina. Galton [49] describes two foramina in the exoccipial-ophistotic of *Dryosaurus*, suggesting they both support two branches of c.n. XII, as later suggested for *Dysalotosaurus* [43] and *Haya* [9], thus it is possible that a branching of c.n. XII is a basal condition for Ornithopoda. The two anterior foramina support the vagus nerve (c.n. X) and the glossopharyngeal nerve (c.n. IX). C.n. X is the second largest foramen in the bone, and due to its size, it might represent the *foramen metoticum* for c.n. X, the accessory nerve (c.n. XI) and the *vena jugularis interna* [26, 49]. Unlike in *Thescelosaurus neglectus*, the small fourth foramen might serve for c.n. IX, separated from the *foramen metoticum*.

Part of the endosseous labyrintolfactory tracts is segmented inside the prootic and the exoccipital/opisthotic (Fig 12M–12O), even though it is not possible to reconstruct the whole inner ear because of the disarticulation of the elements, and the badly preserved inner structure of the supraoccipital hampers further reconstruction. We could not assemble the pieces of the endosseous labyrinth, therefore our description is limited to the single units. The ascending process is almost vertical from the main body, different from the arched processes in *Liaoceratops* [46] *and Thescelosaurus* [42] and the lateral semicircular canal is slightly oriented dorsally. Like in *Thescelosaurus*, a short process extends rostroventrally towards the foramen for cn VII (Fig 12O) [42]; this process is absent in *Liaoceratops* [46]. The ampulla is thick, but the ventral portion consisting of the endosseous cochlear duct is missing. The posterior semicircular canal describes a wider arch than in *Liaoceratops*, similar to *Dysalotosaurus*.

### Tooth replacement

Ornithischian dinosaurs exhibit a wide array of tooth morphologies, dimensions and arrangements, with an evolutionary trend towards the formation of dental batteries in hadrosaurids and ceratopsians (Chen et al., 2018). In fact, most of the studies about tooth replacement, dental inner anatomy and wear-facet analyses are based on these two clades. In recent years, more studies have been conducted in basal neornithischians, offering new insight into the evolutionary dental pathways, as in *Changchunsaurus* [37], *Fruitadens* [50] and *Liaoceratops* [51]. These investigations are possible when CT scans and/or histological analyses can be performed on the specimens, but the rarity and availability of basal neornithischians usually hamper those investigations. Barrett and Han’s [3] previous description of *Jeholosaurus* focused on the external aspects of the teeth, but recently Hu et al. [38] analyzed, under CT scanning, a series of *Jeholosaurus* skulls to reconstruct the tooth replacement pattern across ontogeny. The pristine preservation of YLSNHM 01942 and the digital modelling allow us to provide yet another specimen to the knowledge on this species’s dental growth, confirming the authors’ results. In YLSNHM 01942 there are seven replacement teeth on the left maxilla, and three on the right. In the lower jaw, nine replacement teeth are visible on the left dentary, and 12 on the right one. In both dentaries, there is one single replacement tooth per alveolus, representing the ancestral state for ornithischians [38], present in *Changchunsaurus* [37] but different from the two erupting replacement teeth in *Liaoceratops* [51]. A second generation of replacement teeth is found in the maxilla and dentary of the subadult *Jeholosaurus* IVPP V15717 [38], suggesting YLSNHM 01942 represents a earlier juvenile stage. Different developmental stages can be observed in YLSNHM 01942, corresponding to the process described recently by Hu et al. [38]. Each replacement tooth is associated with a “replacement foramen” opening on the lingual side of the dentary, as in *Changchunsaurus* [37]. The youngest formed replacement tooth is a small, isolated cusp, initially forming at about halfway the root of the adjacent functioning tooth and corresponding to the apical margin of the central eminence. The following development stages correspond to the formation of a leaf-like crown with well-developed denticles, then of the cingulum, and finally of the root. The pressure of the replacement tooth on the active tooth root starts when the crown is almost completely developed, forming a slight depression on the lingual side of the active root, even before the cingulum is formed in the replacement tooth. When the root starts to form, the intrusion onto the alveolar socket follows a diagonal plane, causing the resorption of the root that results in a thin, labiolingually compressed sheet of bone on the rostrolabial side of the dentary. As in *Changchunsaurus*, the replacement teeth do not invade the pulp chamber of the functional teeth until they completely erupted, as observed in the final stage of tooth replacement.

## Conclusion

The skull YLSNHM 01942 is attributed to *Jeholosaurus shangyuanensis* because of the foramen on the quadratojugal. Other diagnostic characters, such as the ornamentation on the postorbitals and the jugals, are missing because of the juvenile stage of the individual, whilst others are not possible to determine due to the fragmentary nature of the nasal and the caudal portion of the jugal. The segmentation of the skull provided new information on the neurocranial anatomy of the species, especially the full reconstruction of the exoccipital/ophistotic, prootics and the basisphenoid, showing the passages for the cranial nerves and the carotid aorta. We improved the scoring for *J*. *shangyuanensis* in the phylogenetic analysis by Dieudonné et al. [8], removing a redundant character and adding new scorings and taxa by Rotatori et al. [11], Czepiński and Madzia [14] and Augustin et al. [12]. The equal weight tree resulted in a well-resolved topology for the basal members of Ornithopoda, with the Chinese *Changmiania liaoningensis* as the basal-most representative of the clade, followed by *Nanosaurus agilis*, *Jeholosaurus shangyuanensis*, *Changchunsaurus parvus*, *Haya griva* and *Yandusaurus hongheensis*. We recognize the fact that this result does not clarify the early evolution of ornithopods, as other phylogenies -based on different set of characters- provide different scenarios [40]. Nonetheless, the inclusion of *C*. *liaoningensis* and *J*. *shangyuanensis* demonstrate the importance of the Chinese ornithopod fauna to reconstruct the basal relationships within the clade, reinforcing the hypothesis that the clade originated in the Eurasian regions [52].

## Supporting information

S1 Fig Unknwon skull remains. Position of the unknown cranial fragments in YLSNHM 01942 in the current location within the skull (in dorsal view). Scale bars equal 1 cm.
(TIF)

S2 Fig Phylogenetical tree with different scoring for *Ajkaceratops*. Strict consensus tree of the parsimony equal weighting analysis, considering the *Ajkaceratops* specimen as a premaxilla. Here, *Ajkaceratops* is detected within Ornithopoda as a sister taxon of *Changmiania liaoningensis*.
(TIF)

S1 File Character list. List of characters modified from Dieudonné et al. [8].
(DOCX)

S2 File Phylogenetical analysis. Folder containing the .tnt matrix, and the results from the script used to run the analysis, following Rotatori et al. [11].
(ZIP)

## Abbreviations

IVPP: Institute of Vertebrate Paleontology and Paleoanthropology, Beijing, China
NHMUK: Natural History Museum of London, UK
SHN: Sociedade de Historia Natural, Torres Vedras, Portugal
YLSNHM: Yingliang Stone Natural History Museum, Nan’an, China.
