# Supplementary figures and images for: Anatomical description and digital reconstruction of the skull of Jeholosaurus shangyuanensis (Dinosauria, Ornithopoda) from China
Source: PLoS One. 2025 Jan 24;20(1):e0312519. doi: 10.1371/journal.pone.0312519 (PMC11760024; doi:10.1371/journal.pone.0312519)

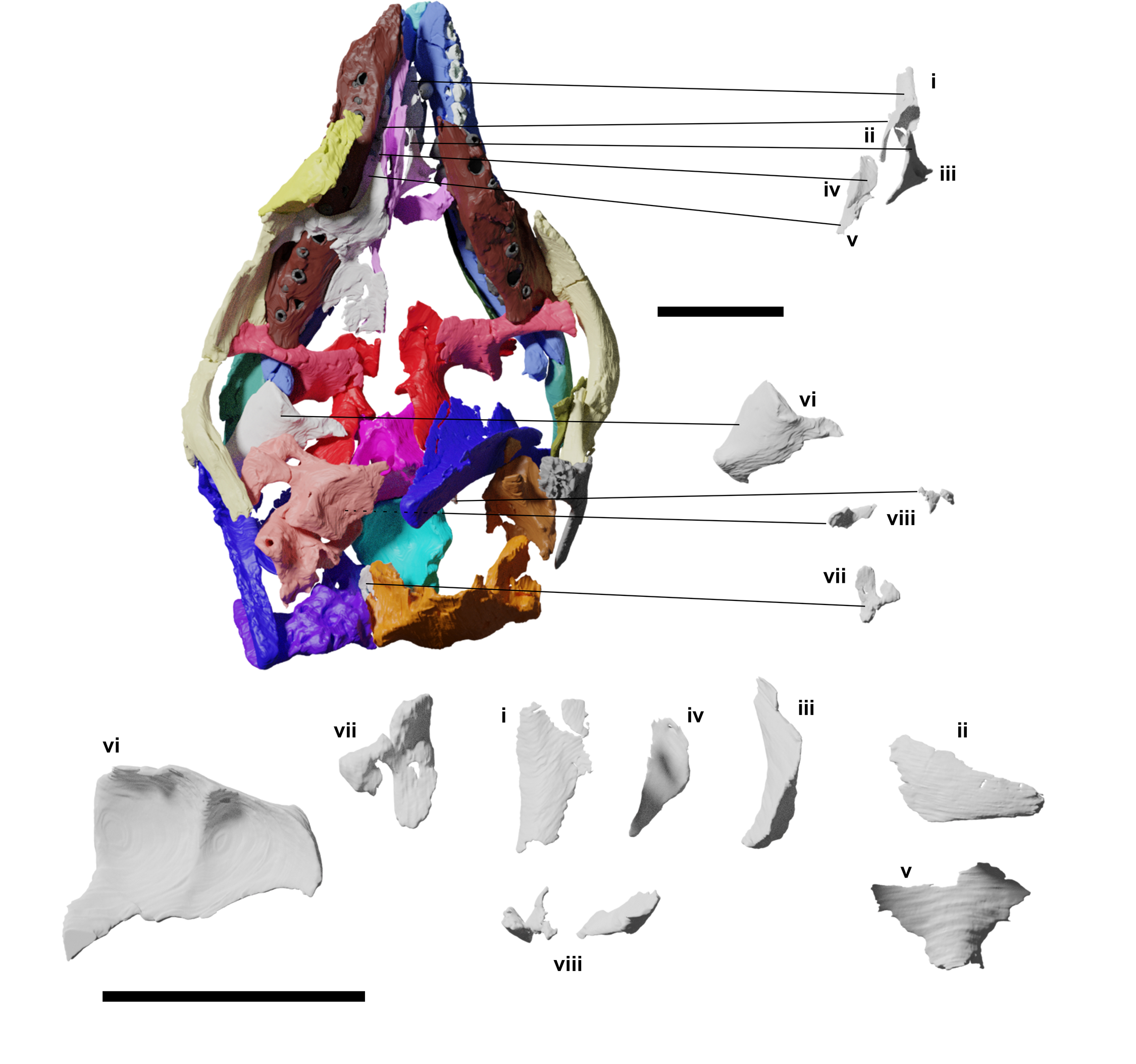

Supplement: S1 Fig — Position of the unknown cranial fragments in YLSNHM 01942 in the current location within the skull (in dorsal view). Scale bars equal 1 cm. (TIF) [file pone.0312519.s001.tif]

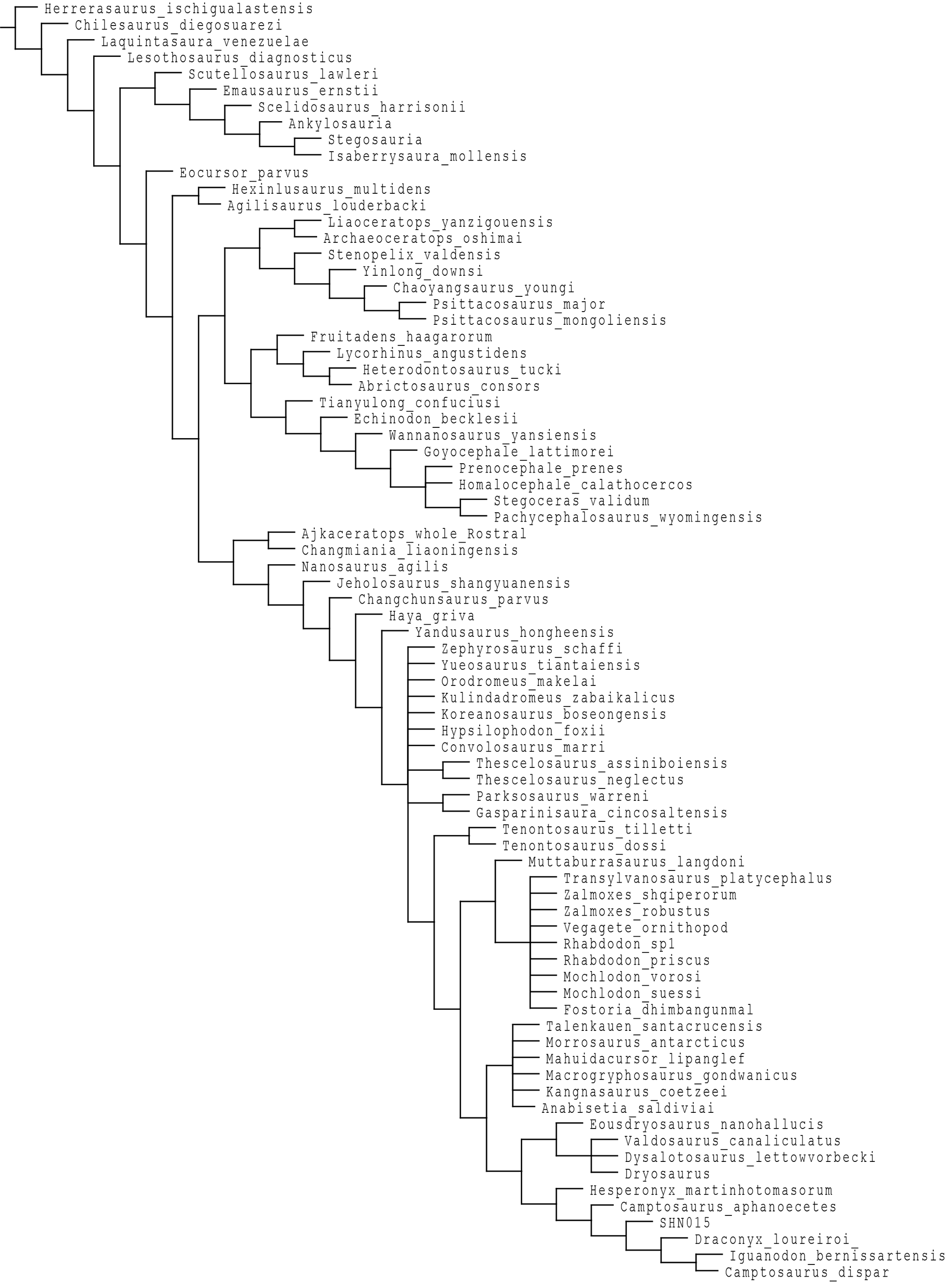

Supplement: S2 Fig — Strict consensus tree of the parsimony equal weighting analysis, considering the Ajkaceratops specimen as a premaxilla. Here, Ajkaceratops is detected within Ornithopoda as a sister taxon of Changmiania liaoningensis. (TIF) [file pone.0312519.s002.tif]
