# Supplementary material for: Anatomical description and digital reconstruction of the skull of Jeholosaurus shangyuanensis (Dinosauria, Ornithopoda) from China
Source: PLoS One. 2025 Jan 24;20(1):e0312519. doi: 10.1371/journal.pone.0312519 (PMC11760024; doi:10.1371/journal.pone.0312519)
Supplement: S1 File — List of characters modified from Dieudonné et al. [8]. (DOCX) [file pone.0312519.s003.docx]

**Character list (Dieudonné et al., 2021)**

1. Skull, rostral-quadrate length relative to the body length: 10 % (0), 13 % or more (1).

2. Skull, preorbital region, percentage out of the total skull length from the rostrum to the quadrate: equal or more than 40% (0), much less than 40% (1).

3. Skull, position of maximum widening of the skull: beneath the jugal–postorbital bar (0), posteriorly, beneath the infratemporal fenestra (1).

4. Lower margin of the infratemporal fenestra with respect to the lower margin of the orbit: level or higher (0), lower (1).

5. Infratemporal fenestra size: small, much smaller than the orbit (0) or large, subequal or larger than the orbit (1).

6. Skull, widening of the skull across the jugals, chord from frontal orbital margin to extremity of jugal is more than minimum interorbital width: absent (0), present, skull has a triangular shape in dorsal view (1)

7. Skull, cortical remodeling of surface of dermal bone: absent (0), present (1)

8. Rostral bone (neomorphic bone anterior to premaxilla): absent (0), present (1)

9. Rostral ventral process: absent (0) or present (1)

10. Rostral, shape of anterior face: round, convex (0) or sharply keeled (1)

11. Rostral bone, ventrolateral processes: rudimentary (0), well-developed (1)

12. Snout, anterior margin shape: sloped posterodorsally, snout shallow (0) or more vertical anterior margin, snout deep (1)

13. Premaxilla, anterior and dorsal surface: lacks rugosities (0), bears distinct rugose surface (1)

14. Premaxilla, ventral inflection: absent, oral margin even with ventral margin of maxilla (0), present, oral margin projects farther ventrally than ventral margin of maxilla (1)

15. Premaxilla, denticles on oral margin: absent (0), present (1)

16. Premaxilla, edentulous anterior region: absent, first premaxillary tooth is positioned adjacent to the symphysis (0), present: if any, the first premaxillary tooth is inset the width of one or more crowns (1)

17. Premaxilla, posterolateral process: does not contact lacrimal (0), contacts the lacrimal, excludes maxilla–nasal contact (1)

18. Premaxilla, ventral (or oral) margin: narial portion of the body of the premaxilla slopes steeply from the external naris to the oral margin (0), ventral premaxilla flares laterally to form a partial floor of the narial fossa (1)

19. Premaxilla, premaxillary foramen: absent (0), present (1)

20. Premaxilla, premaxillary palate: strongly arched, forming a deep, concave palate (0), horizontal or only gently arched (1)

21. Premaxillae: unfused (0), fused (1)

22. Premaxilla, external naris size: small, entirely overlies the premaxilla (0), enlarged, extends posteriorly to overlie the maxilla (1)

23. Premaxillary internarial bar: present, reaches the nasal (0), incomplete or absent (1)

24. Premaxilla, position of the ventral margin of external nares: below the ventral margin of the orbits (0), above the ventral margin of the orbits (1)

25. Premaxilla, narial fossa surrounding external nares on lateral surface of premaxilla, position of ventral margin of fossa relative to the ventral margin of the premaxilla: closely approaches the ventral margin of the premaxilla (0), separated by a broad flat margin from the ventral margin of the premaxilla (1)

26. Maximum length of external nares less than 15% basal skull length (0), maximum length of external nares greater than 15% basal skull length (1)

27. Premaxilla-maxilla contact, fossa-like depression positioned on the premaxilla–maxilla boundary: absent (0), present (1)

28. Premaxilla-maxilla diastema: weak to absent, maxillary teeth continue to anterior end of maxilla (0), present, substantial diastema of at least one crown length between maxillary and premaxillary teeth (1)

29. Premaxilla-vomeral head, ventral contact: present (0), absent, ventrally excluded by midline contact between maxillae (1)

30. Premaxilla-prefrontal contact: absent (0) or present (1)

31. Maxilla, prominent anterolateral boss articulates with the medial premaxilla: absent (0), present (1)

32. Maxilla, at least a small prolongation that bulges out in front of the anterior edge of the maxillary ascending process (not considering the ventral premaxillary process): absent (0), present (1)

33. Maxilla, buccal emargination: absent (0), present (1)

34. Maxilla, eminence on the rim of the buccal emargination of the maxilla near the junction with the jugal: absent (0), present (1)

35. External antorbital fenestra, shape (regardless of position): triangular (0), oval or circular (1)

36. Antorbital fenestra, position of the posterior part with respect to the orbit: passes below the orbit (0), next to or anterior to the orbit (1).

37. External antorbital fenestra, exclusion of the jugal from the posteroventral margin by lacrimal–maxilla contact: absent (0), present (1)

38. External antorbital fenestra, maximum diameter: 60% or more of orbital diameter (0), approximately 50% of orbital diameter (1) or very small or absent (2)

39. Internal antorbital fenestra, length relative to skull length: large, generally at least 15 % (0), very much reduced, less than 10% (1), or absent (2)

40. Antorbital fenestra, position: level or higher than the orbit (0), anteroventral to the orbit (1)

41. Prominent horizontal ridge under the antorbital fossa: absent (0) or present (1)

42. Nasals, depression present along sutural line of the bones: absent (0), present (1)

43. Frontal, contacts orbit: along more than 25% of total frontal length (0), less than 25% (1), excluded from orbital margin (2)

44. Frontal, ratio of frontal length to nasal length: greater than 120% (0), between 120% and 60% (1) or less than 60% (2)

45. Frontals, each one are short and broad (0), narrow and elongate (at least twice as long as wide) (1)

46. Frontals arching over orbit from lateral view: present (0), absent, frontals dorsally flattened over orbit (1)

47. Lacrimal-jugal contact: jugal doesn’t, or barely touches lacrimal (0), jugal meets lacrimal with more contact (1)

48. Lacrimal proportions: anteroposteriorly long, the posterior branch expands posteroventrally to form a slight portion of the anteroventral orbital margin (0), anteroposteriorly short, the posterior branch is not expanding posteroventrally (1).

49. Lacrimal-nasal contact: present (0), absent (1).

50. Accessory ossification(s) in the orbit (palpebral/supraorbital): absent (0), present (1)

51. Palpebral/supraorbital: free, projects into orbit from contact with lacrimal/prefrontal (0), incorporated into orbital margin (1)

52. Palpebral, shape in dorsal view: rod-shaped (0), plate-like with wide base (1)

53. Palpebral/supraorbital, number: one (0), two (1) or three (2)

54. Supraorbital(s) horizontal extension across the orbit (whether fused or not to the orbital margin) : contact the postorbital posteriorly (0), does not contact the postorbital, but crosses at least half of the orbit (1), crosses less than half of the orbit (2)

55. Lower margin of the orbit circular (0), lower margin of the orbit subrectangular (1)

56. Depression on lateral surface of postorbital, : absent, the lateral surface is devoid of any pronounced depression, and varies from smoothly concave to smoothly convex over the whole postorbital anteroposteriorly (0); present and well-demarcated, opens posteriorly toward infratemporal fenestra (1); present on the anterior side toward the orbit (2)

57. Squamosal process of postorbital relative to the jugal process: much shorter (0) or subequal or longer (1)

58. Postorbital: inverted ‘L’-shaped (0), triangular and plate like with normal expansion of squamosal process (1), triangular and plate-like with a very short squamosal process (2)

59. Postorbital participation to the lower temporal opening: present (0), postorbital excluded from margin (1)

60. Postorbital-parietal contact: absent (0), very narrow (1), broad (2)

61. Distinctive indentation on posterior cranial midline between the parietals: present (0) or absent (1)

62. Parietal sagittal crest: narrow shelf or sharply defined crest (0) or broad, essentially absent (1)

63. Parietal fenestration: absent (0) or present (1)

64. Parietal, location of posterior margin relative to squamosal: anterior to (0) or level with or posterior to (1) that of squamosal

65. Parietal, posterior margin relative to rest of skull: below or level with the anterior skull roof (0) higher than the anterior skull roof (1)

66. Squamosal-Quadratojugal contact: present, between dorsal process of quadratojugal and descending process of the squamosal (0), absent (1)

67. Supratemporal fenestra length relative to the basal skull length (BSL): short, fenestrae are less than 25% BSL (0), elongated, more than 25% BSL (1)

68. Squamosal with significant dorsolateral overhang above the descending process of quadrate: absent (0) or present (1)

69.Squamosal, height in posterodorsal direction above quadrate cotylus: lateral side relatively low (0); markedly expanded dorsally above the cotylus (1).

70. Squamosal prequadratic process: present, covers the anterodorsal part of the proximal quadrate shaft (0); absent (1).

71. Parietosquamosal shelf, posteromedial process of squamosal: does not overhang the occipital region (0); overhang the occipital region, forms at least a slight dorsal horizontal shelf (1); consists of a vertically oriented sheet of bone (2)

72. Postorbital-squamosal tubercle/node row: absent (0), present (1)

73. Postorbital-squamosal tubercle row, enlarged tubercle row on the posterior squamosal: absent (0), present (1)

74. Postorbital-squamosal bar, morphology of the ventral junction with the jugal process of postorbital: smoothly concave and large (0); narrow “T-shaped”, sharply angled (1).

75. Squamosal, morphology of postorbital process dorsal to M. adductor mandibulae externus origin site: gently convex (0), mediolaterally compressed and blade-like (1)

76. Jugal with prominent ventral flange: absent (0) or present (1)

77. Jugal anterior process: shallow and tapered (0) or expanded dorsoventrally (1)

78. Jugal wing (formed by quadratojugal and jugal), height that contact the quadrate: greater than 20% quadrate height (0), less than 20% (1).

79. Jugal wing, degree of anteroposterior overlap of the quadrate shaft (not considering the pterygoid wing): complete, reaches the posterior border of quadrate (0), almost complete, cover more than 50% of quadrate length (1), partial, cover much less than 50% of quadrate length (2)

80. Jugal, ventral extent of the wing formed by the jugal and quadratojugal ends: at or near distal condyles of quadrate (0), above distal condyles (1), well above the distal condyles (2)

81. Jugal, articulation with quadrate: jugal fails to articulate with quadrate (0), jugal articulates with quadrate (1)

82. Posterior maxillary process on the medial side of the jugal: straight to modestly arched medially (0), anteromedially projected and arched (1)

83. Jugal, ectopterygoid articular facet on medial view: consists of a deep groove (0), rounded scar (1)

84. Jugal anterior ramus: dorsoventrally deeper than mediolaterally broad (0), broader than deep (1)

85. Jugal, morphology of portion of maxillary process that overlaps maxilla: tapers at anterior ends of maxillary and lacrimal contacts, with slightly convex ventral margin and slightly concave dorsal margin (0), subrectangular with parallel dorsal and ventral margins (1)

86. No boss present on lateral surface of the jugal (0), presence of a boss or horn on the lateral surface of the jugal (1)

87. Jugal ornamentation: absent (0), or present, nodular (1)

88. Jugal-postorbital bar, anteroposterior width relative to that of the infratemporal fenestra: less expanded (0), equally expanded (1), or anteroposteriorly broader than the infratemporal fenestra (2)

89. Jugal-postorbital joint: elongate scarf joint (0), short butt joint (1)

90. Jugal, form of postorbital process: not expanded dorsally (0), dorsal portion of postorbital process expanded posteriorly (1)

91. Jugal, posterior ramus forking: absent (0), present, incision between processes vary from narrow to more than 45° (1)

92. Jugal, posterior ramus: forms anterior and ventral margin of infratemporal fenestra (0), forms part of posterior margin, expands towards squamosal (1)

93. Jugal-squamosal contact: absent, separated by postorbital (0) or present (1)

94. Jugal–quadratojugal contact: overlapping (0), tongue-and-groove (1)

95. Jugal (or jugal–epijugal), ridge dividing the lateral surface of the jugal into two planes: absent (0), present (1)

96. Quadratojugal, shape: inverted L-shaped, with elongate anterior and ventral processes (0), subrectangular with long axis vertical, short, deep anterior process (1), horizontal T-shaped, with sharp angle between the anterior and dorsal processes (2)

97. Paraquadratic/quadratojugal foramen, pierces the quadratojugal: absent (0), present (1)

98. Paraquadratic/quadratojugal foramen size: small and/or narrow if dorsoventrally tall (0) large (1)

99. Paraquadratic/quadratojugal foramen or notch, location: opens between quadratojugal and quadrate, notches the anterior margin of the quadrate (0), opens inside the quadratojugal (1)

100. Quadrate foramen location and orientation of the opening: posteriorly onto the posterolateral aspect of quadrate shaft (0), on lateral aspect of quadrate or quadratojugal (1)

101. Body of the quadrate leans posteriorly (0), body of quadrate oriented vertically (1), body of quadrate leans anteriorly (2)

102. Quadrate, prominent oval fossa on pterygoid ramus: absent (0), present (1)

103. Quadrate, mandibular articulation: quadrate condyles subequal in size (0), medial condyle is larger than lateral condyle (1), lateral condyle is larger than medial (2)

104. Laterosphenoid, socket for the head: occurs along frontal-postorbital suture (0), only in postorbital (1)

105. Post-temporal foramen position: at the boundary between the parietals/squamosals and the paroccipital process (0), entirely within the opisthotic (1), positioned entirely within the squamosal (2)

106. Opisthotic, presence of a ‘Y-shaped’ indentation on the dorsal edge for the passage of the post-temporal foramen: absent (0), present (1)

107. Prootic, position of the foramen for the trigeminal nerve (V): notches the posteroventral edge of the laterosphenoid at the boundary with the prootic (0), nearly or completely enclosed in prootic (1)

108. Prootic-basisphenoid plate: absent (0), present (1)

109. Supraoccipital, contribution to dorsal margin of the foramen magnum: forms entire dorsal margin of foramen magnum (0), exoccipital with medial process that restricts the contribution of the supraoccipital (1), the exoccipital join medially and excludes totally the supraoccipital from the dorsal margin of the foramen magnum (2)

110. Supraoccipital: nuchal crest is present (0) or absent (1)

111. Supraoccipital (SO), anteroposterior inclination and sutural contact with the opisthotics (OP) from a posterior view: the SO is obliquely inclined anteroposteriorly, the sutural contact with the OP is diagonal from the top laterally to the foramen magnum ventromedially (0), the SO completely roofs the endocranial cavity, it overlain the OP and its sutural contact with the OP is horizontal from a posterior view (1), the SO is held almost vertically and sutures nearly vertically with the adjacent opisthotics (2).

112. Paroccipital processes (Exoccipital-Opisthotic complex): extend laterally and transit smoothly toward a slight dorsoventral expansion distally (0), distal end pendent, sharply deflects ventrally (1)

113. Paroccipital processes, proportions: short and deep (height ≥ 1/2 length) (0), elongate and narrow (1)

114. Basioccipital, contribution to the border of the foramen magnum: foramen magnum occupies less than 50% of occipital condyle or is completely excluded from it by the exoccipitals (0), more than 50% of occipital condyle (1)

115. Basioccipital, ventral margin of occipital condyle: forms a neck before a being posteroventrally expanded (0), smooth and continuous, devoid of ventral neck (1)

116. Basioccipital, anteroposteriorly directed groove extending along ventral surface: absent (0), present (1)

117. Basioccipital, median ridge extending along ventral surface: absent (0), present (1)

118. Basioccipital, anteroventral part: produces an elongated process that is “locked” between the basal tubera of the basisphenoid (0), forms a broadly concavo-convex contact with the basisphenoid, or is completely restricted to the posterior aspect of the basal tubera (1).

119. Basioccipital, basal tubera: extend much farther ventrally than the basisphenoid/parasphenoid plate (0), level (1)

120. Basioccipital, basal tubera: level with the base of the basioccipital condyle (0), form a massive buttress which extends much lower than the base of the basioccipital condyle (1)

121. Angle between the base of the braincase (i.e. the axis formed by the occipital condyle and basisphenoid) and long axis of the braincase (i.e. the axis followed by the ventral margin of laterosphenoid): less than 35 degrees (0), equal or more than 35 degrees (1)

122. Basisphenoid, basipterygoid processes articular facet orientation: anteroventral and/or anterolateral (0), ventral (1), posteroventral (2)

123. Notch between posteroventral edge of basisphenoid and base of basipterygoid process: deep (0) or notch shallow with base of basipterygoid process close to basioccipital tubera (1)

124. Basisphenoid, length relative to basioccipital length: longer or subequal (0), shorter than basioccipital (1)

125. Pterygoid, contact with its counterpart: absent, the basicranium is mostly exposed in ventral view, (0) present, the basicranium is mostly obscured in ventral view by an interpterygoid contact formed by the palatal and/or quadrate rami (1)

126. Palatal keel, dorsoventrally deep (deeper than 50% of snout depth) median palatal keel formed of the vomers, pterygoids and palatines: absent (0), present (1)

127. Pterygoid-maxilla contact, at posterior end of tooth row: absent (0), present (1)

128. Pterygoid participation to the pterygo-palatine fenestra: present (0), absent, the ectopterygoid prevents the pterygoid from participating to the pterygo-palatine fenestra (1).

129. Lower jaw, length of post-coronoid elements (from the dorsal border of the coronoid-surangular suture) relative to the total length of the lower jaw: more than 35 (0), 25-35% (1)

130. Predentary: absent (0), present (1)

131. Predentary, size and position: short and the posterior extremity is posteriorly set, the predentary oppose only the first half of the premaxilla (0), short, the posterior border is anteriorly set, all but the posterodorsal corner of the predentary is positioned anterior to the last premaxillary tooth (1), roughly equal in length to the premaxilla, premaxillary teeth only oppose predentary all along (2).

132. Predentary, shape: rounded tip (0), pointed tip (1).

133. Predentary, grooves on either side of midline on anterior surface, extending ventrolaterally to dorsomedially: absent (0), present (1)

134. Predentary, oral margin: relatively smooth (0), denticulate (1)

135. Predentary, tip of in lateral view: does not project above the main body (0), strongly upturned relative to main body (1)

136. Predentary, ventral process: single (0), bilobate (1)

137. Predentary, ventral process: present, well-developed (0), very reduced or absent (1)

138. Predentary length of lateral processes relative to the ventral process: short (0), long, more than half the length of the ventral process (1)

139. Dentary, ratio of dentary height (just anterior to the rising coronoid process) divided by length of dentary: between 15-20% (0), 20-35% (1)

140. Dentary, symphysis: unexpanded (0), V-shaped (1), spout-shaped (2)

141. Dentary, position of the anterior tip: positioned high (0), mid height (1), near lower margin of dentary (2), below lower margin (3)

142. Dentary, morphology of ventral margin of anterior ramus leading to the predentary articulation: straight with an anterior break in slope leading to the tip of the predentary process (0), inflected ventrally before reaching the predentary articulation and symphysis (1), curves in a regular and continuous way dorsally toward the symphysis and predentary articulation (2)

143. Dentary, tooth row (and edentulous anterior portion) in lateral view: straight (0), anterior end downturned (1)

144. Dentary, dorsal and ventral margins before their locking into the predentary: converge anteriorly (0), subparallel (1), deepen anteriorly (2)

145. Dentary, ventral flange: absent (0), present (1)

146. Dentary, orientation of tooth row relative to lateral surface of dentary: convergent anteriorly and posteriorly, bowed medially at mid-length, the tooth row ends anterior and aligned to the coronoid process (0), convergent anteriorly and divergent posteriorly so that the tooth row ends anterior to the coronoid and medial to its longitudinal axis (1), the dentary tooth row ends posteromedially to the coronoid (2)

147. Dentary, coronoid process: absent or weak, posterodorsally oblique, depth of mandible at coronoid is less than 150% depth of mandible beneath tooth row (0), well-developed, distinctly elevated, depth of mandible at coronoid is more than 150% depth of mandible beneath tooth row (1)

148. Dentary, the posterolateral surface bears a profound circular depression: absent (0), present (1)

149. Dentary, number of dentary teeth: 10 or fewer (0), 11–13 (1), 14–17 (2), more than 18 (3)

150. Coronoid, swells ventrolaterally until below the dentary tooth row: abent (0), present (1).

151. Surangular lateral surface: flat to weakly convex (0) or with pronounced laterally convex curvature (1)

152. Ridge or process on lateral surface of surangular, anterior to jaw suture: incipient or absent (0); anteroposteriorly extended ridge (1); dorsally directed finger-like process or strongly bulging boss (2)

153. External mandibular fenestra, situated on dentary-surangular-angular boundary: present (0), absent (1)

154. Retroarticular process: long, subequal to or exceeding the length of the glenoid (0), rudimentary or absent (1)

155. Dentary-angular, node-like ornamentation: absent (0), present (1)

156. Dorsoventral extension of the angular at the level of the coronoid process: forms less than half of the dorsoventral height of the mandibular ramus (0); forms half or more of the height of the mandibular ramus, but remains below the dentary tooth row (1), reach the dorsal extent of the mandibular ramus or is higher (2)

157. Jaw, level of jaw joint: level with tooth row, or weakly depressed ventrally (0), strongly depressed ventrally, more than 40% of the height of the quadrate is below the level of the maxillary occlusal margin (1)

158. Premaxillary teeth: more than three (0), ≤ three (1), absent, premaxilla edentulous (2)

159. Premaxillary (non-caniniform) tooth crown orientations in lateral view: recurved (0) or straight (1)

160. Premaxillary teeth, crown mesiodistal expansion above root: absent, no distinction between root and crown is observable (0), crown is moderately expanded above root (1)

161. Premaxillary teeth, shape: transversely compressed (0), bulbous, strongly convex labially (1)

162. Premaxillary teeth, posterior increase in size (breadth and/or height) : absent, all premaxillary teeth are subequal in size and not significantly broader than the succeeding maxillary teeth (0), premaxillary teeth increase in breadth and height posteriorly, and the most posterior tooth is larger than succeeding maxillary teeth (1)

163. Premaxillary teeth, denticles: present (0), absent (1).

164. Premaxillary tooth row and anterior portion of maxillary tooth row: aligned with each other (0), maxillary teeth are inset the width of one or more crowns from the premaxillary teeth (1)

165. Teeth, crown mesiodistal expansion above root in cheek teeth: very weak to absent (0), present (1)

166. Teeth, close-packing and quicker replacement eliminating spaces between alveolar border and crowns of adjacent functional teeth: absent (0), present (1)

167. Teeth, wear facets on teeth: absent or sporadically developed (0), systematic development of wear facets along the entire tooth row (1)

168. Maxillary/dentary teeth, position of maximum apicobasal crown height in tooth rows: anterior portion of tooth row (0), central portion of tooth rows (1), posterior portion of tooth rows (2)

169. Maxillary/dentary teeth, marginal ornamentations: fine serrations set at right angles to the margin of the tooth (0), coarse serrations (denticles) angle upwards at 45 degrees from the margin of the tooth (1)

170. Maxillary/dentary teeth, enamel symmetrical (0), asymmetrical (1)

171. Maxillary teeth, number and morphology of secondary/accessory ridges on labial surface of crown: no secondary ridges, only accessory ridges or swellings arising from marginal denticles (0), a few parallel and apicobasally extending secondary and accessory ridges (1), multiple parallel and apicobasally extending secondary and accessory ridges so that entire labial surface is corrugated (2)

172. Maxillary/dentary teeth crown from a labial view, mesiodistal borders extend into a prominent mesial and distal bounding ridge and terminate in an apical denticle: absent (0), present (1)

173. Maxillary/dentary teeth, at least weakly developed labiolingual expansion of the crown (‘cingulum’) above the root: absent (0), present (1)

174. Maxillary/dentary teeth, interdental space: non-packed teeth (0), lack of space between adjacent teeth up through the occlusional margin (1), overlapping of adjacent crowns with an overlapping “en échelon” pattern (2)

175. Maxillary/dentary alveolar foramina (‘special foramina’) medial to tooth rows: present (0), absent (1)

176. Maxillary teeth, crown shape: lingually concave (0), lingually convex (1)

177. Maxillary and dentary tooth crowns, apicobasal height: high, ratio of crown height / maximum mesiodistal width ≥ 1.5 (0); low, ratio < 1.5 (1)

178. Maxillary and dentary teeth, cingulum height and shape (with the usually associated crown shape): cingulum very low and crown triangular (0), cingulum moderately high, ‘U-shaped’ and crown spade-like or triangular (1), cingulum high with a V-shaped vertex, and diamond-shaped crowns (2)

179. Maxillary teeth, apical ridge or swelling position, centrally placed (0), posteriorly set (1)

180. Maxillary tooth crown, mesiodistal edges: diverging from the root (0), chisel-shaped with parallel sides (1)

181. Maxillary teeth, relative prominence of the primary ridge on labial surface of crown: primary and secondary ridges absent or weakly developed from the apex of the crown (0), outstanding in comparison to other secondary ridges (1), completely undistinguishable from at least a few other secondary ridges in (2)

182. Maxillary teeth, root shape: straight (0), curved (1)

183. Dentary dentition, heterodonty: no substantial heterodonty is present in dentary dentition (0), single, enlarged, caniform anterior dentary tooth (1)

184. Dentary teeth, peg-like tooth located anteriorly within dentary lacks denticles, strongly reduced in size: absent (0), present (1)

185. Dentary teeth, intercrown spaces: present (0), absent (1)

186. Dentary teeth, apical ridge position: anteriorly or centrally positioned (0), posteriorly positioned (1)

187. Dentary teeth, number of ridges reaching the base of the crown: fewer than 10 (0), more than 10, and often more than 17 (1)

188. Dentary teeth, shape and prominence of the primary ridge on lingual surface of the crown: absent, there is a smooth swelling instead of a primary ridge (0), the primary ridge is mesiodistally as thin as the secondary ridges and varyingly deep labiolingually (1), the primary ridge largely oversizes secondary ridges in both height and width, and also oversizes all the maxillary teeth ridges (2)

189. Dentary teeth, number and morphology of secondary and/or accessory ridges on lingual surface of the crown: no secondary ridges, faint accessory ridges arising from marginal denticles (0), multiple parallel and evenly-spaced secondary ridges on either side of the central ridge, such that entire lingual surface is corrugated (1), a few parallel and well defined secondary ridges with multiple faint accessory ridges arising from marginal denticles (2)

190. Ridges present on both the labial and lingual sides of dentary crowns (0), ridges mostly limited to the lingual side of dentary crowns and very faint to absent on the labial side (1)

191. Dentary teeth, root shape in cross-section: round (0), oval (1), squared (2)

192. Dentary tooth roots straight in anterior or posterior view all along the row (0), dentary tooth roots curved in anterior or posterior view (1)

Axial skeleton:

193. Cervical vertebrae, shape of postzygapophyses: posterodorsally arched and higher dorsoventrally (0), dorsally flat and dorsoventrally low (1)

194. Cervical vertebrae, heightening of neural spines along the series: remains low and triangular all along, more than three times as long as high in posterior cervicals (0), reach a substantial height posteriorly, less than twice as long as they are tall (1)

195. Axis neural spine: anteroposteriorly short (0), long, extends caudally to overlap more than half of the total length of C3 cervical centrum (1)

196. Postaxial cervical vertebrae, epipophyses on the postzygapophyses somewhere within the neck: present (0), absent (1)

197. Cervical vertebrae (4-9), form of central surfaces: amphicoelous (0), at least slightly opisthocoelous (1)

198. Ventral surface of the cervical vertebrae rounded (0), presence of a broad, flattened keel on the ventral surface of the cervical vertebrae (1), presence of a sharp ventral keel on the ventral surface of the cervical vertebrae (2)

199. Anterior cervical centra less than 1.5 times longer than tall (0), length of anterior cervical centra equal or greater than 1.5 times longer than tall (1)

200. Cervical vertebrae, evolution of central length throughout the series: central length remains approximately the same or decrease posteriorly (0), increase posteriorly (1).

201. Cervical vertebrae, number: 7/8 (0), 9 (1), 10 or more (2)

202. Dorsal vertebrae, number: 12–13 (0), 14-15 (1), 16 or more (2)

203. Dorsal vertebrae, neural spine: anteriorly positioned or centered over the dorsal centrum (0), start projecting farther posteriorly than their own centra at some point within the dorsal vertebral series (1)

204. Sacrum composed of three or fewer fused vertebral centra (0), sacrum composed of between four and five fused vertebral centra (1), sacrum composed of six fused vertebral centra (2), sacrum composed of seven or more fused vertebra centra (3)

205. Sacral vertebrae, neural spines height: less than 2 times the height of the centrum (0), neural spines between 2 and 2,5 times the height of the centrum (1), greater than 2,5 times (2)

206. Sacrum, accessory articulation with pubis: pubis does not articulate with the sacrum (0), pubis supported by sacral rib (1), pubis supported by sacral centrum (2)

207. Ischiac peduncle of the ilium is not supported by a sacral rib (0), ischiac peduncle of the ilium supported by a sacral rib (1)

208. Proximal caudal vertebrae, neural spines position: caudal neural spines positioned over centrum (0), project backward beyond own centrum to an angle of more than 50° over the horizontal (1), project backward to an angle of less than 50° over the horizontal (2)

209. Anterior caudal vertebrae, neural spines: height (from above the prezygapophyses) the same or up to 50% taller than the centrum (0), more than 50% taller than the centrum (1)

210. Dorsal ribs, transition between a near vertical orientation of the tuberculum and capitulum to a horizontal orientation: occurs within ribs 2-4 (0), 5-6 (1), 6-8 (2)

211. Anterior dorsal ribs, distal portions of the shaft in cross-section: circular or oval (0), highly laterally compressed with concave lateral and rugose posterior surfaces (1)

212. Dorsal ribs, distal anteroposterior thickening: absent (0), present (1).

213. Ossified epaxial tendons along dorsal and sacral vertebrae: absent (0), present (1).

214. Partial ossification of the sternal segments of the cranial dorsal ribs absent (0), present (1)

215. Proximal caudal ribs, location: borne on centrum (0), on neurocentral suture (1), on neural arch (2)

216. Caudal ribs, longest rib position: the first caudal vertebra bears longest rib (0), longest rib posterior to the first (1)

217. Distal caudal chevrons shape: rod-shaped, often with slight distal expansion (0), strongly asymmetrically expanded distally (1)

218. Ossified epaxial/hypaxial tendons along caudal vertebrae: absent (0), present (1)

219. Ossified epaxial tendons (back or tail), arrangement: longitudinally arranged (0), Basket-like arrangement of fusiform tendons in caudal region (1), double-layered lattice (2).

Appendicular skeleton:

220. Ossified clavicles: absent (0), present (1)

221. Scapula-Humerus, proportions: scapula longer or subequal to the humerus (0), humerus substantially longer than the scapula (1)

222. Scapula, blade-shape: strongly expanded distally (0), weakly expanded, near parallel-sided (1)

223. Scapula, scapular blade length relative to minimum width: relatively short and broad, length is 5-8 times minimum width (0), elongate and strap-like, length is at least 9 times the minimum width (1)

224. Scapula, acromion shape: weakly developed or absent (0), well-expanded anteriorly, spine-like (1)

225. Scapula, acromion process proximal extent: low, almost reaches the coracoid anterodorsally (0), high, elevated with respect to the coracoid (1).

226. Scapula, angle formed by the medial borders of the ‘supra-glenoid’ process: acute, less than 75° (0), more than 75° (1)

227. Scapula, posterior edge of the supra-glenoid process: smoothly deflects posteroventrally with respect to the ventral edge of the scapular shaft (0), sharply deflects posteroventrally with respect to the ventral edge of the scapular shaft (1)

228. Coracoid, height divided by length (considering an horizontal inclination of the scapulocoracoid, and by omitting the “extra-height” induced by the sternal process with respect to the infraglenoid corner): between 70% and 120% (0) equal or greater than 120% (1)

229. Coracoid, coracoid foramen position from a lateral view: enclosed within coracoid (0), open along coracoid-scapula suture (1)

230. Coracoid, development of the sternal process: short and broad (0), extremely elongated and narrow (ratio greater than 0.80) (1)

231. Sternal plates, shape: absent (0), kidney-shaped or semi-lunate (1), shafted or hatchet-shaped (rod-like posterolateral process, expanded anteromedial end) (2), of right-angle triangle with broad medial contact for collateral sternal (3)

232. Humerus, length relative to femoral length: more than 60% (0), less than 60% (1)

233. Humerus, appearance of the anterior surface in proximal view: a variably developed flexor bicipital sulcus is visible (0), the anterior surface is straight to smoothly convex, no bicipital sulcus visible (1)

234. Humerus, convergence between lateral and medial edges of the proximal extremity in anterior/posterior view: very smooth, the lateral border between head and deltopectoral crest is straight or gently convex (0), very strong, the lateral edge can become concave between the head and the deltopectoral crest (1)

235. Humerus, proximal head separated from prominent medial tubercle on proximal surface by a shallow median groove: absent (0), present (1)

236. Humerus, deltopectoral crest: well developped, projecting at a distinct angle from the shaft (0), low and rounded (1), almost imperceptible (2)

237. Humerus, deltopectoral crest shape: distal margin rounded and merges gradually with the lateral margin of the humeral shaft (0), distal margin angular and merges abruptly with the lateral margin of the humeral shaft (1)

238. Humerus, proximolateral margin with respect to the main axis of the shaft in anteroposterior view: straight, aligned with the distolateral margin (0), medially bowed (1)

239. Humerus, proximal shaft curvature from a lateral view: proximal portion is aligned with distal portion of the shaft (0), proximal portion of the humeral shaft is bent backward relative to the distal portion (1).

240. Humerus, anterior coronoid fossa from a distal view: more deeply incised (0), widely open and shallow (1).

241. Humerus, posterior olecranon fossa from a distal view: present (0), forms only a weak depression or is totally absent (1).

242. Ulna, olecranon process: low (0), moderately developed (1), high (2)

243. Ulna, distal end: directed ventrally in medial or lateral view (0), curves gently posteriorly in medial or lateral view (1)

244. Radius, distal end: subspherical to ovate (0), anteroposteriorly expanded, with its medial surface sub-parallel to closely juxtaposed to the ulna (1), mediolaterally more expanded than the ulna, the radius expands distally at right angle from the ulna and does not cross over it (2).

245. Carpus, fusion: unfused (0), fused (1)

246. Ulnare, cushion-like and proximodistally compressed in dorsoventral view: absent (0), present (1).

247. Ulnare: articulates distally via the distal carpal 4: with the third metacarpal (0), mostly with the fourth metacarpal (1).

248. Metacarpals, block-like proximal ends: absent (0), present (1)

249. Metacarpals I and V: substantially shorter in length than metacarpal III (0), subequal in length to metacarpal III (1)

250. Metacarpal I greater than 50% the length of metacarpal II (0), metacarpal I less than 50% the length of metacarpal II (1)

251. Metacarpal/manual phalanges, extensor pits on the dorsal surface of the distal end: absent or poorly developed (0), deep, well-developed (1)

252. First finger phalanx of digit I, length relative to the first finger phalanx of digit III: significantly longer (0), subequal or shorter (1).

253. First finger phalanx of digit II, length relative to the first finger phalanx of digit III: significantly longer (0), subequal or shorter (1).

254. Penultimate phalanx of fingers II and III: shorter than or subequal to first phalanx (0), longer than the first phalanx (1)

255. Manual digit III, number of phalanges: 4 (0), 3 or fewer (1)

256. Manual unguals, strongly recurved with prominent flexor tubercle: absent (0), present (1)

257. Ilium length taken from the tip of the preacetabular process to the tip of the postacetabular process (measured on a straight line with a ruler): shorter than (0), or longer than (1) 90% of the femur length

258. Ilium postacetabular process from a lateral view: much deeper than (0) or subequal to (1) that of the preacetabular process

259. Ilium, preacetabular process shape and length: short, tab-shaped, distal end is posterior to pubic peduncle (0), elongate, strap-shaped, distal end is anterior to pubic peduncle (1)

260. Ilium, preacetabular process length relative to the ilium length: less than 50% (0), more than 50% (1)

261. Ilium, preacetabular process curvature from a lateral view: no distinct break in slope, dorsal surface varies from straight to smoothly convex all along (0), the downward break in slope located above the pubic peduncle (1), the downward break in slope starting well anterior to the pubic peduncle (2).

262. Ilium, outline of dorsal margin from a dorsal view: postacetabular process straight until above the acetabulum, and the preacetabular process subtly to moderately deflected from midline laterally (0), the dorsal margin forms a regular and continuous curve from the postacetabular process to the preacetabular process, with the medial side convex all along and the preacetabular process well deflected laterally (1), sigmoidal: the preacetabular process is well deflected laterally, and the postacetabular process curves toward the medial side posteriorly (2), straight all along (3)

263. Ilium, preacetabular process expands mediolaterally towards its distal end in dorsal view: absent (0), present (1)

264. Ilium, medioventral acetabular flange, partially closing the acetabulum: present (0), absent (1)

265. Ilium, supra-acetabular ‘crest’ or ‘flange’ on the dorsolateral part of the acetabulum: present (0), absent (1)

266. Ilium, postacetabular process orientation from a lateral view: posteriorly directed (0), curves posterodorsally with both its dorsal and ventral margins (1)

267. Ilium, morphology of dorsal margin at the level of the acetabulum: smooth, almost no modification of dorsal margin (0), well thickened above the ischiac peduncle onward (1), thickened above the pubic peduncle onward (2)

268. Ilium, laterally-bulging everted rim on the dorsal margin above the acetabulum: absent (0), present (1)

269. Ilium, dorsal surface of postacetabular process until the origin of M. iliocaudalis from a lateral view: smoothly convex with a posterior break in slope (0), the postacetabular blade looks strongly quadrangular-shaped (1), tapers with no break in slope for the attachment of M. iliocaudalis (2)

270. Ilium, brevis shelf and fossa: faces ventrolaterally and shelf is near vertical and creates a deep postacetabular portion anteriorly (0), fossa faces ventrally for most of its length and is less visible from a lateral view (1), the brevis shelf consists in a small and smooth ridge that is only visible from a medial view (2)

271. Ilium, brevis shelf and fossa, transverse width: narrow (0); very broad and expanding in width towards its caudal margin such that it appears triangular in dorsal or ventral view (1) (McDonald, 2012 #132).

272. Ilium, length of the postacetabular process relative to the total ilium length: 20% or less (0), 25-35% (1), more than 35% (2)

273. Ilium, pubic peduncle: elongate and robust (0), ventrally projected, elongate and strap-like (1), often reduced in size, anteriorly projected so its distal tip is higher than the ventral extent of the ischial peduncle (2)

274. Ilium, ischiac peduncle: projects ventrally (0), broadly swollen, projects ventrolaterally (1)

275. Ilium, ischiac peduncle: anteroposteriorly short (0), massive and anteroposteriorly long (1).

276. Ilium, acetabulum: normal to high (0), low (1)

277. Pubis, massive and dorsolaterally rotated body obscuring the obturator foramen in lateral view: absent (0), present (1)

278. Pubis, orientation: anteroventral (0), rotated posteroventrally to lie alongside the ischium (opisthopubic) (1)

279. Pubis, prepubic process: absent (0), present (1)

280. Pubis, prepubic process shape in its distal extremity: compressed mediolaterally, dorsoventral height exceeds mediolateral width (0), rod-like, mediolateral width exceeds dorsoventral height (1), dorsoventrally compressed (2)

281. Pubis, prepubic process length: stub-like and poorly developed, extends only a short distance anterior to the pubic peduncle of the ilium (0), elongated into distinct anterior process, but does not extend beyond the distal end of the preacetabular process of ilium (1), elongate and extending up to the level or beyond the distal end of preacetabular process of ilium (2)

282. Pubis, angle between prepubic process and distal postpubic shaft: less than 130 degrees (0) ; greater than 130 degrees but less than 170° (1) ; aligned along the same plane (2)

283. Pubis, pubic symphysis: elongate or at least present distally on a significant part of the pubic blade (0), much reduced or absent (1)

284. Pubis, shape of the postpubis shaft in cross-section: blade-shaped (0), rod-shaped (1)

285. Pubis, length of postpubis shaft relative to ischium length: approximately equal (0), extends for around half the length (1), very short to absent (2)

286. Ischium, pubic peduncle shape: transversely compressed (0), dorsoventrally compressed and mediolaterally thick (1)

287. Ischium, pubic peduncle breadth from a lateromedial view: larger than or subequal to that of the iliac peduncle (0); much smaller than that of the iliac peduncle (1)

288. Ischium, orientation of the proximal main axis of the shaft and angle with respect to the pubic peduncle: falls between the iliac and pubic peduncles main axis, angle inferior to 140° (0), falls between the iliac and pubic peduncles main axis, angle widely open and superior to 140° (1), falls in the same axis of that of the pubic peduncle (2)

289. Ischium, curvature of the acetabular margin in lateral view: gentle, defines a wide acetabular recess (0), marked, defines a narrow acetabular recess (1) (Gasca et al. 2014 #4).

290. Ischium, angle formed by the posterior margin of the iliac peduncle and the posterior margin of the proximal ischial shaft: superior to 120° (0), equal or inferior to 120° (1)

291. Ischium, tab-shaped obturator process: absent, lacks an obturator process (0), present and placed 60% down the shaft of ischium (1), placed within the first proximal half of the shaft (2)

292. Ischium, shaft in cross-section: compressed mediolaterally (0), subcircular and bar-like (1)

293. Ischium, symphysis length: median symphysis with the opposing blade along at least 50% of its length (0), symphysis only presents distally (1)

294. Femur, shape in medial/lateral view: bowed anteriorly along length (0), straight (1)

295. Femur, femoral head: arises from a well-constricted neck (0), arises from a shallow and thick, unconstricted neck (1).

296. Femur, femoral head: confluent with greater trochanter, fossa trochanteris consists in a smooth and shallow groove (0), fossa trochanteris is modified into distinct constriction separating head and greater trochanter (1)

297. Femur, anterior extension of the greater trochanter beyond the femoral head: almost inexistent (0), shortly expanded and thick anteriorly (1), moderately to very elongated (2)

298. Anterior trochanter, level with respect to the greater trochanter: well below (0), from moderately below to slightly below the level of the greater trochanter (1) level or higher (2)

299. Anterior trochanter of femur in proximal view: positioned anterior to greater trochanter (0), possess a beveled posterior surface (anteromedial to posterolateral in direction), so that it appears positioned somewhat anterolateral to the greater trochanter (1), L-shaped anterior trochanter with very thin edges bordering both anterior and lateral sides of the greater trochanter (2)

300. Posterolateral edge of the greater trochanter: globular and rounded (0), triangular, the lateral edge of the greater trochanter is globally flattened (1)

301. Femur, fourth trochanter shape: low eminence or absent (0), straight ridge (1), pendent (2)

302. Femur, fourth trochanter position: located entirely on proximal half of femur (0) or positioned at mid-length, or distal to mid-length (1)

303. Femur, pendent fourth trochanter, rod-like with subparallel anterior and posterior surfaces: absent (0), present (1)

304. Femur, location of insertion scar of M. caudifemoralis longus: extends from fourth trochanter onto medial surface of femoral shaft (0), widely separated from fourth trochanter, restricted to medial surface of femoral shaft (1)

305. Femur, anterior (extensor) intercondylar groove: absent (0), shallow and wide open through with sides that diverge from each other cranially (1), deep and narrow open through with parallel sides (2)

306. Femur, posterior (flexor) intercondylar groove: fully open (0), medial condyle inflated laterally, partially covers opening of flexor groove (1)

307. Femur, posterolateral condyle position and size in ventral view: positioned relatively laterally and slightly narrower in width than the medial condyle (0), strongly inset medially, reduced in width relative to medial condyle (1)

308. Femur, cranial expansion of medial condyle: equal to, or less than lateral condyle (0), protrudes cranially to lateral condyle, and continues onto the cranial surface as a diaphyseal ridge to cranial trochanter (1) (Herne, 2014 #233).

309. Femur proportions in distal view by taking the iliofibularis groove as a reference point whenever possible or the posterior intercondylar groove in all other cases: maximum anteroposterior length of the distolateral condyle (without considering the posterolateral condylid) out of distal width: ≥ 50% (0), between 40 and 50% (1), < 40% (2).

310. Tibia, lateral fibular condyle from an anteroposterior view: gradually and merges with the shaft distally (0), defines an abrupt overhanging buttress with sub-horizontal ventral margin above the shaft (1).

311. Tibia, cnemial crest from a proximal view: straight, faces anteriorly (0), strongly bent laterally (1).

312. Tibia, distal shape: subquadrate, posterolateral process not substantially developed (0), elongate posterolateral process, backing fibula (1)

313. Tibia, maximum expansion of distal end relative to proximal: distal end is considerably less expanded than proximal (0), maximum expansion of distal end is subequal or larger than that of proximal end (1)

314. Fibula, proximal head: moderately expanded at both sides (0), features a major anterior expansion of its anteroproximal corner (1).

315. Fibula, shaft in cross-section: elliptical or round (0), D-shaped (1)

316. Fibula, distal end is strongly reduced and splint-like: absent (0), present (1)

317. Astragalus/calcaneum, indistinguishable and fused to one another: absent (0), present (1)

318. Astragalus, anterior process: moderate to high, from tooth-like to wide anteriorly (0), low to absent (1)

319. Astragalus, posterior side size: low (0), high (1)

320. Astragalus, fibular facet on the lateral margin of the proximal surface: large (0), reduced to small articulation or absent (1)

321. Calcaneum, tibial articular surface from a lateral view: facet for tibia absent (0), facet for tibia present and subequal in length to that for the fibula (1), facet for tibia longer than the facet for the fibula, and the posteroventral part of the calcaneum is elongated into a distinct caudal process (2)

322. Calcaneum, angle between the edge separating the tibial and fibular articular facets, and the lateral border of the calcaneum on the posterior side: greater than 110 degrees (0), less than 110 degrees (1)

323. Medial distal tarsal, shape: blocky in dorsal view (0), thin and rectangular (1), round (2)

324. Medial distal tarsal: articulates distally with metatarsal III only (0), articulates distally with metatarsals II and III (1)

325. Lateral distal tarsal, shape in dorsoventral view: square (0), kidney-shaped (1), sub-triangular (2)

326. Metatarsal II/metatarsal III, morphology of the proximal contact: continuous, flat to smoothly concave anteroposteriorly (0), metatarsal II forms a lateral step over a proximal outgrowth on the ventro-medial side of the metatarsal III (1)

327. Metatarsal II, width of proximal articular surface at mid dorsoplantar height (at the level of its lateral “step” whenever present): inferior to 75% the maximum width of MT III (0), exceeds 75%, but is still below 100% of MT III maximum width (1), equals or exceeds 100% the maximum width of MT III (2).

328. Metatarsal III, dominance of proximal articular surface, width of MT III largely exceeds width of metatarsal IV (by omitting the eventual posteromedial process of MT IV and associated caudolateral notch on MT III): absent (0), present (1)

329. Metatarsal III and IV proximal contact, dorsolateral notch on the proximolateral surface of MT III for eventual dorsomedial overlap of metatarsal IV: absent (0), present (1).

330. Metatarsal IV, proximal extremity: sends a prominent posteromedial process toward MT III, which is eventually hosted within a deep caudolateral notch on MT III: absent (0); present (1)

331. Metatarsal III and IV, proximal contact: tightly adpressed, no notch is observed posteriorly between them (0), conspicuous concavity to either, or both, the posterolateral side of metatarsal III and the posteromedial side of metatarsal IV which can eventually host the fifth metatarsal (1)

332. Metatarsal V, length relative to that of metatarsal III: more than 50% (0), less than 25% (1)

333. Metatarsal V: bears digits (0), lacks digits (1)

334. Metatarsal I, proximal surface: developed into a distinct articular surface (0), proximally splint-like or devoid of any articular surface (1).

335. Pedal digit I, number of pedal phalanges on the first metatarsal: two phalanges (0), bears only one ungual or does not bear digits at all (1)

336. Pedal digit I, configuration: the first metatarsal is well-developed, distal end of last phalanx projects beyond the distal end of metatarsal II (0), metatarsal I reduced or absent, end of phalanx I-1 not extending beyond the end of metatarsal II (1)

337. Pedal unguals, shape: tapering, narrow pointed, claw-like (0), wide, blunt, hoof-like (1)

Dermal skeleton

338. Mandibular osteoderm: absent (0), present (1)

339. Dermal osteoderms, parasagittal row on the dorsum of the body: absent (0), present (1)

340. Dermal osteoderms, lateral row of keeled dermal osteoderms on the dorsum of the body: absent (0), present (1)

341. Dermal osteoderms, U-shaped cervical/pectoral collars composed of contiguous keeled osteoderms: absent (0), present (1)
